# Fused toes homolog, a potential molecular regulator of human papillomavirus type 16 E6 and E7 oncoproteins in cervical cancer

**DOI:** 10.1371/journal.pone.0266532

**Published:** 2022-04-14

**Authors:** Prabakaran D. S., Pankaj Kumar Chaturvedi, Dineshkumar Krishnamoorthy, Young-Seok Seo, Mallikarjuna Thippana, Woo-Yoon Park

**Affiliations:** 1 Department of Radiation Oncology, Chungbuk National University Hospital, Cheongju, Republic of Korea; 2 Department of Plant Science, School of Biological Sciences, Central University of Kerala, Kerala, India; 3 Department of Biotechnology and Bioinformatics, School of Life Sciences, University of Hyderabad, Gachibowli, Hyderabad, India; 4 Department of Radiation Oncology, Chungbuk National University College of Medicine, Cheongju, Republic of Korea; Karpagam Academy of Higher Education, INDIA

## Abstract

Human papillomavirus type 16 (HPV16) plays a major role in the development of cervical cancer. The oncogenic potential of HPV16 is attributed to E6 and E7 oncoproteins. Here, we investigated the relationship between fused toes homolog (FTS) and HPV16 E6 and E7 in cervical cancer cells. HPV16-positive CaSki and SiHa cell lines were used for *in vitro* studies. FTS silencing was performed using a small interfering RNA (siRNA)-based approach, and western blotting was performed to determine the protein expression of tumor suppressors and cell survival markers. Immunoprecipitation, immunofluorescence, *in silico* analysis, and immunohistochemistry were performed to determine the interaction between, and intracellular co-localization of, FTS and both the E6 and E7 proteins. Silencing of FTS reduced the expression of the E6 and E7 proteins in cervical cancer cell lines and conversely increased the expression of the tumor suppressor proteins p53 and retinoblastoma protein. However, the primary transcripts of HPV16 E6 and E7 were unaffected by FTS silencing; furthermore, FTS transcription was unaffected by silencing of either E6 or E7, suggesting their interaction occurs post-translationally. Immunofluorescence and immunohistochemistry analysis demonstrated co-localization of FTS with the HPV16 E6 and E7 proteins, while immunoprecipitation results suggested that FTS interacts with both E6 and E7. Furthermore, *in silico* structural analysis identified putative residues involved in the binding of FTS with E6 and E7. Taken together, these results show that FTS affects both HPV16 E6 and E7 oncogenes in cervical cancer. We propose FTS as a target for the prevention of cervical cancer development and progression.

## Introduction

Human papillomavirus (HPV) is a small, circular, double-stranded DNA virus, with subtypes that are broadly classified into low-risk and high-risk types. The low-risk HPVs induce only benign genital warts, whereas high-risk HPVs are associated with cancers of the genitalia, including the uterine cervix, and of the oropharynx [1]. Oncogenic HPV subtypes, such as HPV 16 and 18, are most frequently associated with human cervical cancer [1]. The HPV genome has three distinct regions: the early gene coding region (E), late gene coding region, and long control region. The early genes (E1, E2, and E4-E7) encode regulatory and viral replication proteins; of these, E5, E6, and E7 are oncogenic [2]. While all the encoded genes are crucial for viral replication and the life cycle, the E6 and E7 oncogenes play a critical role in cervical cancer initiation and progression. These two driving oncogenes take over the cellular ubiquitin-proteasome system (UPS) to degrade the tumor suppressors p53 and retinoblastoma protein (pRb). Degradation of pRb forces the host cell into S-phase, where replication machinery and other resources, such as nucleotide pools, are abundant; taken together, these factors facilitate viral replication [3]. Although the impromptu progression of the cell cycle triggers apoptosis, E6 concurrently degrades p53, leading to evasion of apoptosis, unrestrained cell division, and genomic instability [4]. Thus, these events offer cells a growth advantage and stimulate oncogenesis. HPV16 E6 and E7 also augment trophoblastic growth and intensify the malignant phenotype by impairing cell adhesion, leading to increased cellular motility, invasive properties, and radiation resistance [5, 6]. Furthermore, the two oncogenes upregulate matrix metalloproteinase 2 (MMP-2) and matrix metalloproteinase 9 (MMP-9) and induce epithelial-mesenchymal transition (EMT) transcription factors in cervical cancer and head and neck squamous cell carcinoma (HNSCC) [7, 8]. p53 and pRb are the most extensively studied cellular targets of the E6 and E7 oncogenes, respectively. However, recent reports have demonstrated that these two viral oncogenes have the potential to target many more cellular factors, including epigenetic regulators. These downstream factors could exert global changes that could eventually culminate in uncontrolled cell proliferation and carcinogenesis [9].

The fused toes homolog (FTS) gene was initially identified by Lesche et al. as one of the six genes deleted in a mouse mutant known as fused toes [10]. Previously, we reported that FTS expression increases gradually in the progressive grades of cervical cancer, from cervical intraepithelial neoplastic (CIN) lesions I to CIN III, and negatively regulates the tumor suppressor protein, p21 [11]. We have also reported a role for FTS in radio resistance and cisplatin chemoresistance in cervical cancer [12, 13]. In the current study, we evaluated the regulatory effects of FTS on HPV16 E6 and E7 in cervical cancer.

## Materials and methods

### Cell culture and reagents

HPV16-positive cervical cancer cell lines, CaSki (cat. no. 21550) and SiHa (cat. no. 30035), were recently obtained from the Korean Cell Line Bank (KCLB; Seoul, Korea). The cell lines were validated by short tandem repeat profiling, carried out by the KCLB. The CaSki cells were maintained as monolayer cultures in Roswell Park Memorial Institute-1640 (cat. no. LM 011–01, Welgene, Daegu, Korea), while SiHa cells were grown in Dulbecco’s Modified Eagle’s Medium (cat. no. LM 001–05, Welgene) supplemented with 10% fetal bovine serum (FBS; cat. no. S 001–07, Welgene), 100 units/mL penicillin, and 100 μg/mL streptomycin (cat. no. LS 202–02, Welgene), and incubated in a humidified atmosphere at 37°C with 5% CO_2_. Phenol-free trypsin-ethylene diamine tetra acetic acid solution (cat. no. LS 015–08, Welgene) was used for routine passaging and cell harvesting. All primary and secondary antibodies were purchased from Santa Cruz Biotechnology (Dallas, TX, USA), unless otherwise stated.

### Immunoblot analysis

Cells cultured in 90 mm dishes (cat. no. 20100, SPL Life Sciences, Pocheon, Korea) were washed with ice-cold Dulbecco’s phosphate-buffered saline, pH 7.4 (D-PBS; cat. no. LB 001–02, Welgene), and collected using a cell scraper (cat. no. 90021, SPL Life Sciences). Cells were then lysed using 250 μL of radioimmunoprecipitation assay lysis buffer (cat. no. 9806, Cell Signaling Technology Inc., Danvers, MA, USA), incubating for 30 min on ice. Cell lysates were collected into microcentrifuge tubes (cat. no. 60115, SPL Life Sciences) and centrifuged at 10,000 x g at 4°C (Micro 17R, Hanil Scientific Inc., Gimpo, Korea). The supernatants were collected and stored at -70°C until analysis. The protein concentration was measured colorimetrically using Bradford reagent (cat. no. 500–0006, Bio-Rad, Hercules, CA, USA), and 25 μg of total protein from each sample was resolved on 4–20% Tris-Glycine gels (cat. no. XP04205BOX, Invitrogen, Carlsbad, CA, USA). The resolved proteins were then transferred onto polyvinylidene difluoride membranes (cat. no. IPVH00010, Carrigtwohill, Ireland) and probed with the appropriate primary and secondary antibodies. The blots were developed using an enhanced chemiluminescent substrate (cat. no. 34577, ThermoFisher Scientific, Rockford, IL, USA) and visualized using a Luminescent Image Analyzer (LAS 3000 mini, Fujifilm, Tokyo, Japan).

### Silencing of FTS, HPV16 E6, and HPV16 E7

Cells were treated with FTS small interfering RNA (siRNA; final concentration: 50 nM) to achieve FTS silencing, whereas control cells were treated with scrambled siRNA. FTS siRNA (cat. no. sc-93013, Santa Cruz) is a cocktail of 3 target specific 19–25 nucleotide sequences designed to knock down gene expression of FTS, whereas the control siRNA (control siRNA cat. no. sc-37007, Santa Cruz) contains a scrambled sequence that will not lead to the knockdown of any known cellular mRNA. Silencing of HPV16 E6 and E7 gene expression was performed using siRNAs to target the respective viral mRNAs and the control groups were treated with scrambled siRNA (HPV16 E6: cat. no. sc-156008; HPV16 E7: cat. no. sc-270423; control siRNA cat. no. sc-37007; Santa Cruz). Briefly, cells were grown overnight in an antibiotic-free media. The following day, the media was replaced with a transfection media (cat. no. sc-36868, Santa Cruz) containing an appropriate amount of respective siRNAs, along with transfection reagent (cat. no. sc-29528, Santa Cruz). After incubation at 37°C for 6 h, the media was then replaced with complete growth media, and cells incubated for a further 24 h to achieve gene silencing.

### Total RNA extraction and reverse transcription polymerase chain reaction (RT-PCR)

After transfection with control or FTS siRNA for 24 h, cells were washed with ice-cold PBS (cat. no. ML 008–01, Welgene). Total RNA was extracted from CaSki and SiHa cells using TRIzol reagent (cat. no. 15596–026, Invitrogen), according to the manufacturer’s protocol, and cDNA was synthesized with oligo-dT primers using the Omniscript RT kit (cat. no. 205113, Qiagen, Hilden, Germany), as described by the manufacturer. Glyceraldehyde 3-phosphate dehydrogenase (*GAPDH*; human) was used as an internal control to calculate relative expression. The expression of HPV16 E6, E7, FTS, and *GAPDH* genes, was assessed using appropriate primers, as described previously [14, 15], in FTS-, E6-, or E7-silenced and control cells. The primer sequences are as follows: HPV16 E6–5’-GAGAACTGCAATGTTTCAGGAC-3’ and 5’-CCACCGACCCCTTATATTATGG-3’; HPV16 E7–5’-GCAACCAGAGACAACTGATCTCTAC-3’ and 5’-GGTCTTCCAAAGTACGAATGTCTACG-3’; FTS—5’-CACTGGGGTGAGGCTTACTGCC-3’ and 5’-TGGCTGCACATAGACGCCTGG-3’; GAPDH—5’-GTCCGAGTCACCGCCTGCCG-3’ and 5’-CTCGGCTGGCGACGCAAAAG-3’. The PCR was performed on T100 thermal cycler (Bio-Rad) and involved an initial denaturation step at 95°C for 3 min, followed by 36 cycles of 95°C for 20 s, 30 s at the optimal primer annealing temperature for each gene (E6 and E7: 54°C; FTS: 65°C; GAPDH: 52°C), and primer extension at 72°C for 30 s, with a final extension at 72°C for 2 min. The PCR products (5.0 μL) were then directly loaded onto 2% agarose gels (cat. no. C-9100, Bioneer, Daejon, Korea) alongside a DNA size marker (to confirm the amplified products were of the predicted size; cat. no. D-1030, Bioneer), and stained with RedSafe (cat. no. 21141, iNtRON Biotechnology, Seongnam, Korea). Gels were visualized and analyzed using a Gel Doc XR+ (Bio-Rad). Each experiment was performed in three independent repeats.

### Immunofluorescence (IF)

Cells were plated onto coverslips (cat. no. 0111520, Paul Marienfeld, Lauda-Königshofen, Germany) and allowed to attach. Subsequently, they were transfected with control siRNA (Santa Cruz) or FTS siRNA (Santa Cruz). After 24 h, cells were then fixed with 4% paraformaldehyde (cat. no. J19943 1 LT, Thermo Scientific, Geel, Belgium) for 20 min and permeabilized with 0.1% Triton X-100 (cat. no. 0694-1L, Amresco, Solon, OH, USA) in D-PBS for 15 min at room temperature (RT). This was followed by overnight incubation at 4°C with anti-FTS (cat. no. sc-134343, Santa Cruz) and either anti-E6 (cat. no. sc-1584, Santa Cruz) or anti-E7 (cat. no. bs-10446R, Bioss, USA) antibodies. All antibodies were diluted 1:100 in 2% FBS/D-PBS. The following morning, cells were washed three times with D-PBS and further incubated for 1 h at RT with Alexa 488- and Alexa 594-conjugated secondary antibodies. Nuclei were counterstained with 1 μg/mL 4’,6-Diamidino-2-phenylindole (cat. no. D8417, Sigma, Saint Louis, MO, USA). Finally, the coverslips were mounted onto slides (cat. no. 1000612, Paul Marienfeld) and dried overnight in the dark. Images of random coverslip regions were captured and analyzed using a confocal laser scanning microscope (LSM 880, Carl Zeiss Microscopy, LLC, White Plains, NY, USA). Pearson’s coefficient for co-localization was calculated using JACoP: Just Another Co-localization Plugin [16] for ImageJ [17].

### Immunoprecipitation (IP) assay

An immunoprecipitation assay was performed as described previously [18]. Briefly, 24 h after treatment with FTS siRNA, cells were lysed using cell lysis buffer (cat. no. 9803S, Cell Signaling Technology) for 30 min on ice. Lysates were centrifuged at 10,000 x g for 10 min at 4°C, and the supernatants collected. A total of 200 μg protein from each sample was incubated overnight at 4°C with the anti-FTS antibody (Santa Cruz), followed by incubation for 1 h with Protein A/G PLUS-Agarose (cat. no. sc-2003, Santa Cruz). The immunoprecipitates were washed twice with ice-cold lysis buffer. Bead-bound proteins were eluted with sodium dodecyl sulfate (SDS) loading buffer at 95°C for 3 min, and subjected to SDS-PAGE and western blot analysis for FTS, E6, and E7.

### Immunohistochemistry (IHC)

Cervical cancer tissues (n = 10) were collected at the Department of Pathology, Chungbuk National University Hospital. The tissues were studied according to the procedures approved by the Institutional Review Board (IRB file no. 202111005) of the Chungbuk National University Hospital. The tissues were analysed anonymously, so no consent was required from the participants. All samples had been fixed in formalin and processed into paraffin blocks according to routine surgical pathology practice. Histological evaluation of the cervical carcinoma was performed as described previously [11]. Briefly, 4 μm thick tissue sections were deparaffinized with xylene and rehydrated with a series of graded ethanol solutions before staining. Sections were antigen-retrieved using citrate buffer (cat. no. ab94674, Abcam, Cambridge, MA, USA) in a microwave oven for 2 min, following the manufacturer’s instructions. Endogenous peroxidase was blocked using Dako Dual Endogenous Enzyme Block (cat. no. S2003, Dako, Carpinteria, CA, USA) for 10 min. The Abcam TripleStain IHC Kit (cat. no. ab183290, Abcam) was used to observe co-localization in human tissue sections. The manufacturer’s protocol was slightly modified to visualize co-localization of two proteins. Anti-FTS mouse IgG and either anti-HPV16 E6 goat IgG, or anti-HPV16 E7 rabbit IgG, were added to the slides at dilutions of 1:100, and incubated at 4°C overnight. After washing in 0.1% Tween-20 in D-PBS, the sections were incubated with the respective secondary antibodies for 60 min. The color reaction was developed using the three chromogens contained in the TripleStain Kit: 3,3’-diaminobenzidine (brown, for E6), Permanent Red (red, for E7), and Emerald (green, for FTS), according to the manufacturer’s recommendations. Sections were counterstained with hematoxylin, and dried overnight at RT. The slides were stored at -20°C, and removed only for imaging. Images were captured and analyzed with an Olympus IX71 microscope (Olympus, Tokyo, Japan).

### Molecular modeling, docking, and structural analyses

In the present study, we have identified interacting residues in the protein-protein binding complex by performing molecular docking studies separately between FTS and HPV16 E6, E7 proteins. In order to perform docking studies, we have searched for the three-dimensional (3D) structures of the above-mentioned proteins against the Protein Data Bank (RCSB PDB) it resulted that only the Human papillomavirus type 16 E6 protein (HPV16 E6) has structure in RCSB PDB with PDB ID 4GIZ. FTS and Human papillomavirus type 16 E7 protein (HPV16 E7) doesn’t have solved structure in RCSB PDB. Hence, there is a need to build a 3D model of FTS and HPV16 E7 to proceed further. For this purpose, we retrieved the amino acid sequence of FTS protein from the UniProt database with accession number Q9H8T0 (292 amino acids). Template search has been performed against Protein Data Bank with BLASTp, suggesting that no reliable template is available for FTS. 3D structure of the protein was modeled using the I-TASSER (Iterative Threading ASSEmbly Refinement) [19] webserver, which employs multiple threading approaches and iterative template fragment assembly simulations for generating theoretic structures of query proteins. Models were generated using the top 10 threading templates identified by LOMETS [20], a meta-server threading approach, from the PDB library. Based on high C-score and TM-score models were selected for molecular docking studies. For Human papillomavirus type 16 E7 protein (HPV16 E7), we build a 3D model using GalaxyWEB [21], a protein structure prediction server. We have used the protein sequence available at UniProt with accession number P03129.

Molecular docking studies between HPV16 E6 and FTS, HPV16 E7, and FTS were performed using the GRAMM-X [22] protein-protein docking server. The top-ranked docking complex was analyzed for the interactions between the proteins using PyMol molecular graphics system.

### Statistical analysis

All analytical data are presented as the mean of three independent experiments. Differences between the groups were calculated by unpaired, parametric, two-tailed T-tests, performed using GraphPad Prism version 9.1.0 (221) for Windows (GraphPad Software, La Jolla, CA, USA, www.graphpad.com); *p* ≤ 0.05, was considered statistically significant.

## Results

### FTS silencing reduces the expression of E6 and E7

**Fig 1:**
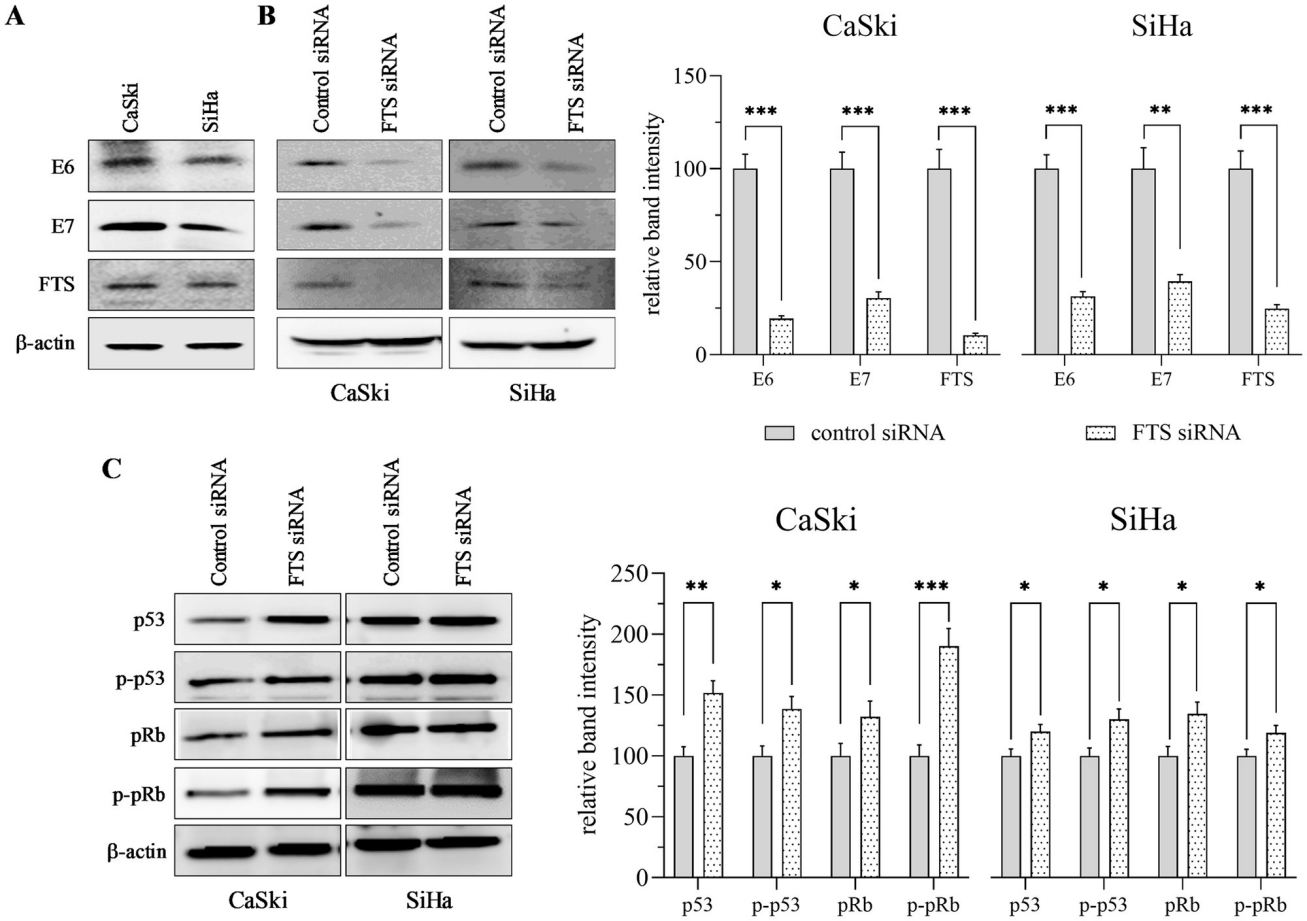
Silencing of FTS reduces HPV16 E6/E7 oncoproteins level and increases tumor suppressor level in CaSki and SiHa cells. Expression of HPV16 E6/E7 and FTS in the cell lines (A). The expression levels of E6 and E7 in the control and FTS silenced group (B). The tumor suppressor proteins p53 and pRb were determined by western blot in the control and FTS silenced group (C). Actin was used as a loading control. The bar graphs adjacent to each blot represents mean ± standard error of the mean (SEM) of densitometric analyses performed on the blots obtained from three independent experiments. The data has been normalized to control. Differences between the groups were calculated by unpaired, parametric, two-tailed T-tests. p-values of statistically significant differences are shown. * p <0.05; ** p < 0.005; *** p < 0.001.

First, we examined the basal expression of FTS, and HPV16 E6 and E7, in CaSki and SiHa cells by western blotting. E6, E7, and FTS were expressed in ample quantities in both the cell lines (Fig 1A). However, the expression levels of all three oncoproteins varied between the two cell lines, with slightly higher expression levels of the oncoproteins found in CaSki cells compared to SiHa cells. To understand the role of FTS in HPV16-mediated cervical cancer, we silenced FTS using an siRNA-based approach. The efficiency of silencing, as validated by a decline in the expression levels of FTS, was approximately 90% in CaSki cells, and 75% in SiHa cells (Fig 1B). Interestingly, densitometric observations identified decreased expression of HPV16 E6 and E7 in CaSki and SiHa cells following FTS silencing. E6 protein levels were reduced by approximately 80% and 70%, while E7 levels were reduced by 70% and 60%, in CaSki and SiHa cells, respectively, as compared to the control group (Fig 1B). Following FTS silencing, we further checked the expression of the tumor suppressor proteins p53 and pRb, which are downstream targets of E6 and E7. The results indicated a considerable increase in p53, p-p53, pRb, and p-pRb protein levels in CaSki cells (50%, 40%, 30%, and 90%, respectively), and a moderate increase in SiHa cells (20%, 30%, 35%, and 19%, respectively; Fig 1C). These results suggest a putative role for FTS in cervical cancer mediated by E6 and E7.

### Silencing of either HPV16 E6 or E7 diminishes FTS expression

**Fig 2:**
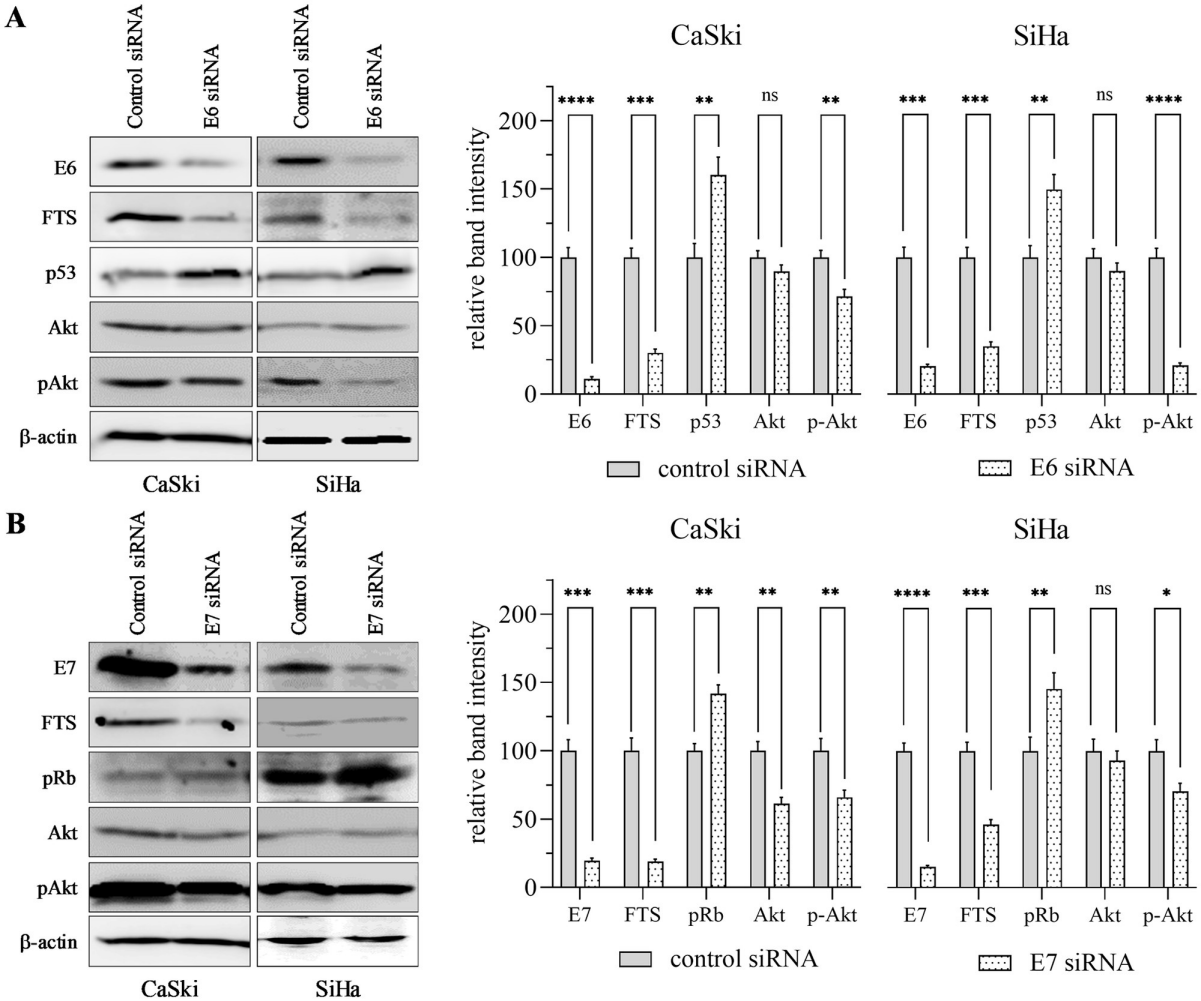
Silencing of HPV16 E6/E7 oncogenes reduces FTS level and increases tumor suppressors level in CaSki and SiHa cells. The levels of tumor suppressor proteins p53, pRb, and cell survival marker Akt and pAKT were determined by western blot in the control and E6 (A) or E7 (B) silenced groups. Actin was used as a loading control. The bar graphs adjacent to each blot represents mean ± SEM of densitometric analyses performed on the blots obtained from three independent experiments. The data has been normalized to control. Differences between the groups were calculated by unpaired, parametric, two-tailed T-tests. p-values of statistically significant differences are shown. * p < 0.05; ** p < 0.01; *** p < 0.001; **** p <0.0001.

To understand the functional role of the HPV16 E6 and E7 oncogenes in the regulation of FTS and its downstream target, Akt, in cervical cancer cells, we transfected CaSki and SiHa cells with E6- and E7-specific siRNA. Reduced expression of FTS and Akt, as well as of phosphorylated-Akt (p-Akt), was observed in both the cell lines. Moreover, as expected, we found increased expression of the tumor suppressors p53 and pRb in E6- and E7-silenced cells, respectively (Fig 2A and 2B). It was intriguing to note that the reduction in E6 and E7 expression induced by FTS silencing could also be seen in the opposite direction, with silencing of E6 and E7 diminishing FTS expression.

### Effects of FTS silencing on HPV16 E6 and E7 transcription, and vice versa

**Fig 3:**
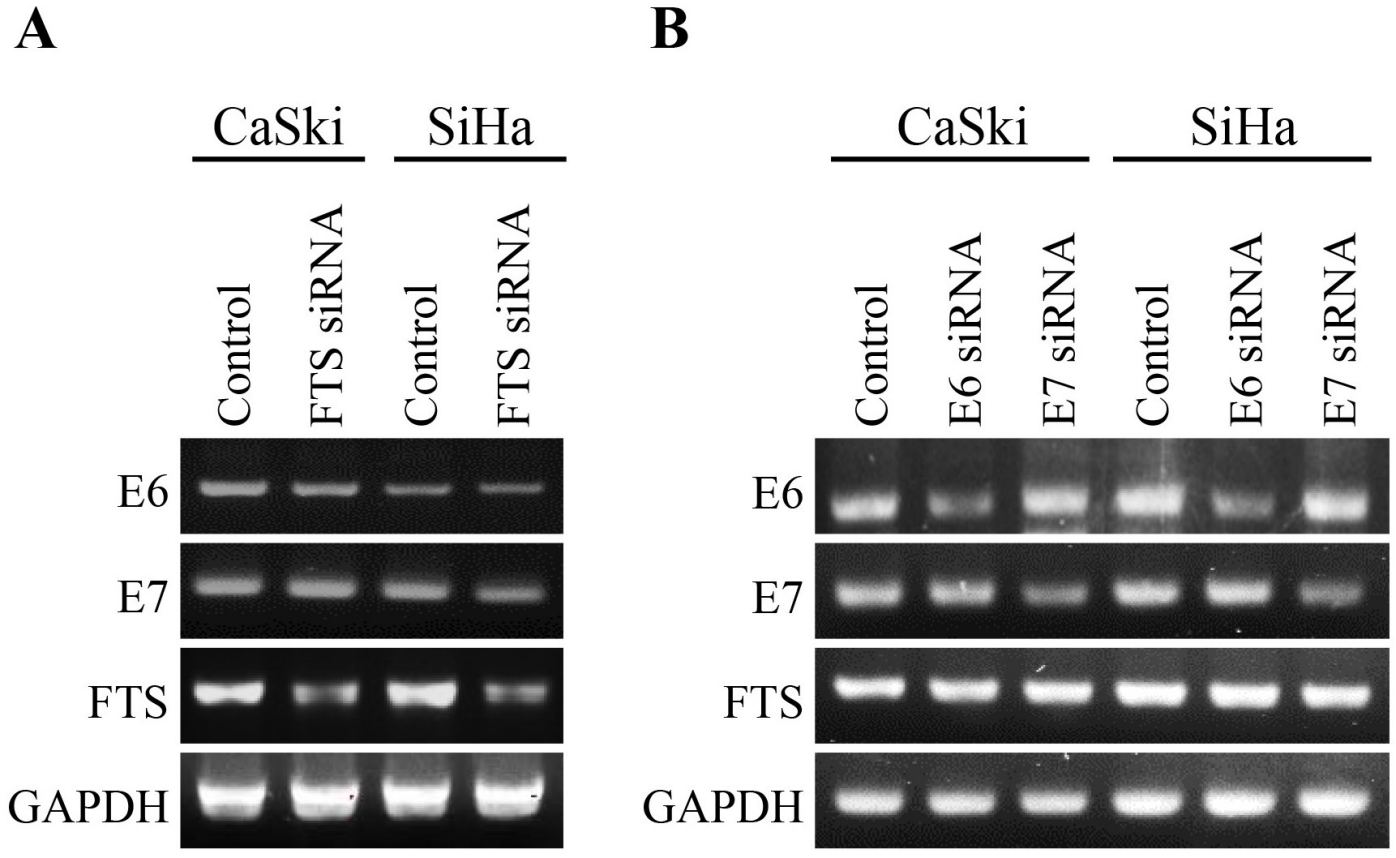
Silencing of FTS has no effect on the transcription of HPV16 E6/E7 oncogenes or *vice-versa* in CaSki and SiHa cells. mRNA expression of HPV16 E6/E7 was evaluated in FTS intact/silenced group (A). Further, FTS mRNA levels were also verified in HPV16 E6/E7 intact/silenced group (B). GAPDH was used as positive control.

To examine the effects of FTS silencing on HPV16 E6 and E7 transcription in CaSki and SiHa cells, and vice versa, mRNA levels were analyzed by RT-PCR (Fig 3). The transcription of HPV16 E6 and E7 remained unchanged upon silencing of FTS. A similar trend was observed for FTS transcripts upon silencing of HPV16 E6 and E7. Therefore, we performed western blot analysis for E6, E7, and FTS, in control and FTS-silenced CaSki and SiHa cells, in the presence or absence of a proteasome inhibitor (MG262), and the translation inhibitor cycloheximide (CHX). It was evident that in FTS-silenced cells, MG262 rescued E6 and E7 proteins by at least 50%, while in the presence of CHX, E6 protein levels were depleted in an hour (S1 and S2 Figs). These observations suggest that neither FTS, nor E6 and E7, interact with the primary transcripts of each other; instead, their interaction is post-translational.

### FTS co-localizes with HPV16 E6 and E7

**Fig 4:**
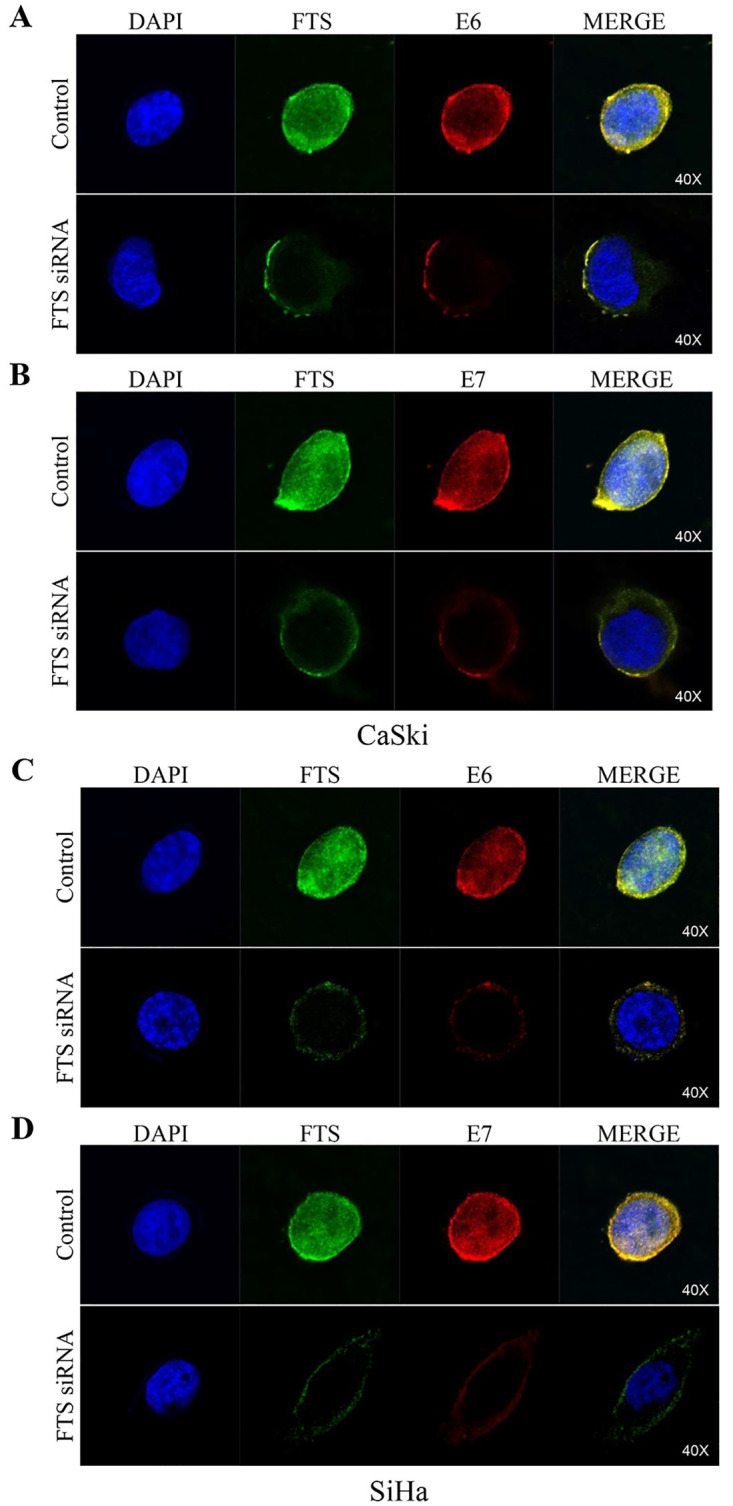
Co-localization of FTS and E6/E7 in FTS silenced cells. Expression of FTS and E6 in CaSki cells (A), FTS and E7 in CaSki cells (B), FTS and E6 in SiHa cells (C), and FTS and E7 in SiHa cells (D). FTS was stained with Alexa Fluor 488 (green), while E6 or E7 was stained with Alexa Fluor 594 (red). The nuclei were counter-stained with DAPI. The Pearson’s coefficient for FTS co-localization with E6/E7 revealed that the co-localization ranged between 0.834–0.938.

IF analysis demonstrated co-localization of FTS with E6 and E7 in the nuclear region. Moreover, silencing of FTS decreased the expression of both cellular oncogenes E6 and E7 (Fig 4). We measured the co-localization of FTS with E6 and E7 using JACoP for ImageJ. The Pearson’s coefficient of correlation ranged between 0.845 and 0.938, where M1 (FTS over E6 and E7) values were in the range of 0.762 to 0.907, while M2 (E6 and E7 over FTS) values ranged from 0.873 to 0.975 (S1 Table), suggesting a very high degree of co-localization. Although the expression of E6 and E7 oncogenes was reduced upon silencing of FTS, the degree of co-localization did not change significantly.

### Interaction between FTS and HPV16 E6 and E7

**Fig 5:**
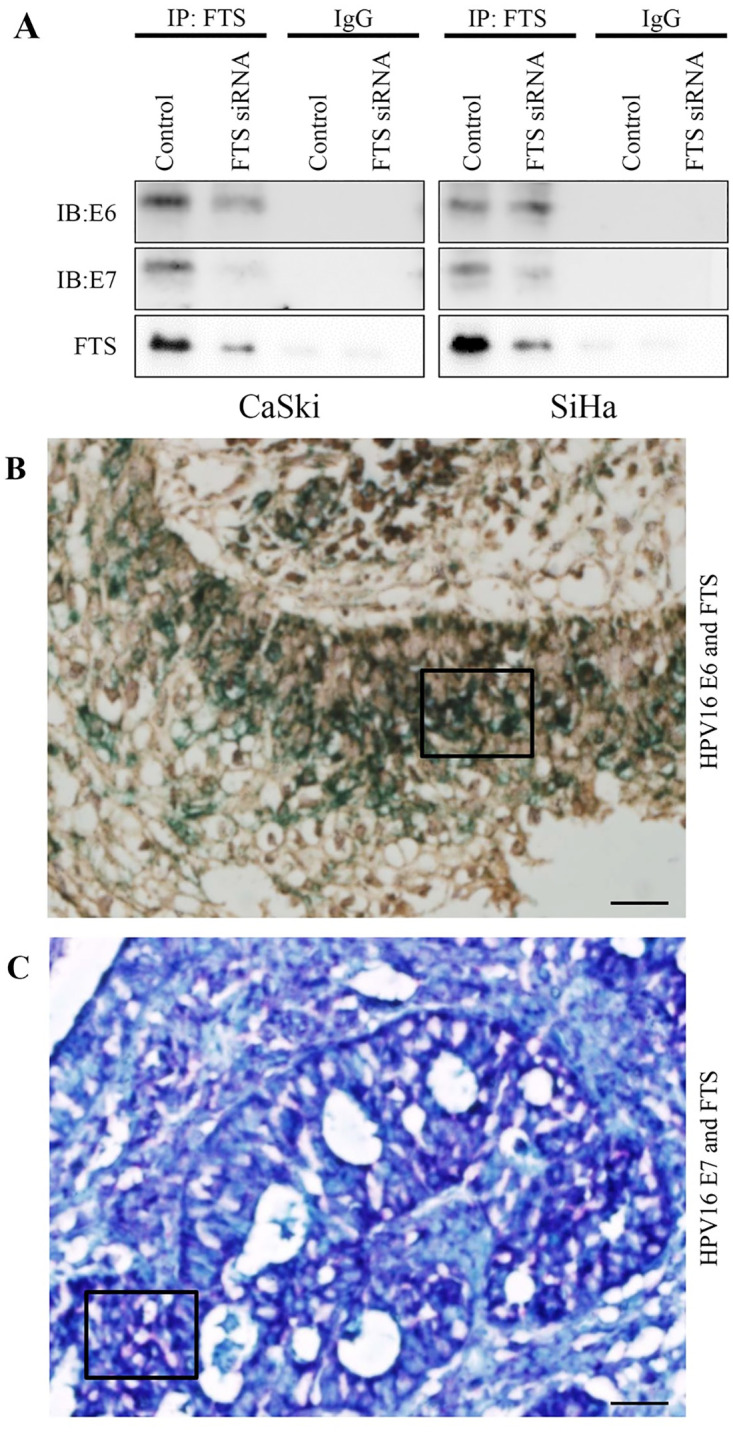
FTS interacts and co-localizes with E6/E7 in cervical cancer cell lines and tissues. IP shows binding between FTS and E6/E7 in CaSki and SiHa cells (A), expression and co-localization of FTS (green color) and HPV16 E6 (brown color) in cervical cancer tissues (B), and expression and co-localization of FTS (green color) and HPV16 E7 (red color) in cervical cancer tissues (C). The co-localized area with maximum intensity is enclosed by a black box. The images of IHC shown are representative of 10 cervical cancer tissues. Black bar in (B) and (C) corresponds to 20 μm.

To further strengthen the findings of the IF assay and confirm the physical association between FTS and the E6 and E7 proteins, we performed an IP assay. The IP results suggested a strong interaction between FTS and HPV16 E6 and E7 in FTS-intact cells, as validated by strong signals in the group treated with control siRNA. However, silencing of FTS prevented these interactions (Fig 5A). Furthermore, to gain insight into these interactions *in vivo*, we examined the co-localization of these oncoproteins by IHC, using a triple staining kit. IHC findings were in complete agreement with the IP and IF assay findings, indicating co-localization of FTS (green) with E6 (brown) and E7 (red). A change in the original stain color of the target molecules was seen in areas of co-localization, e.g., a blackish green color where FTS and E6 co-localized, and a purple color in the areas of FTS and E7 co-localization (Fig 5B and 5C).

### Identification of residues involved in the interaction of FTS with E6 and E7, using molecular docking

**Fig 6:**
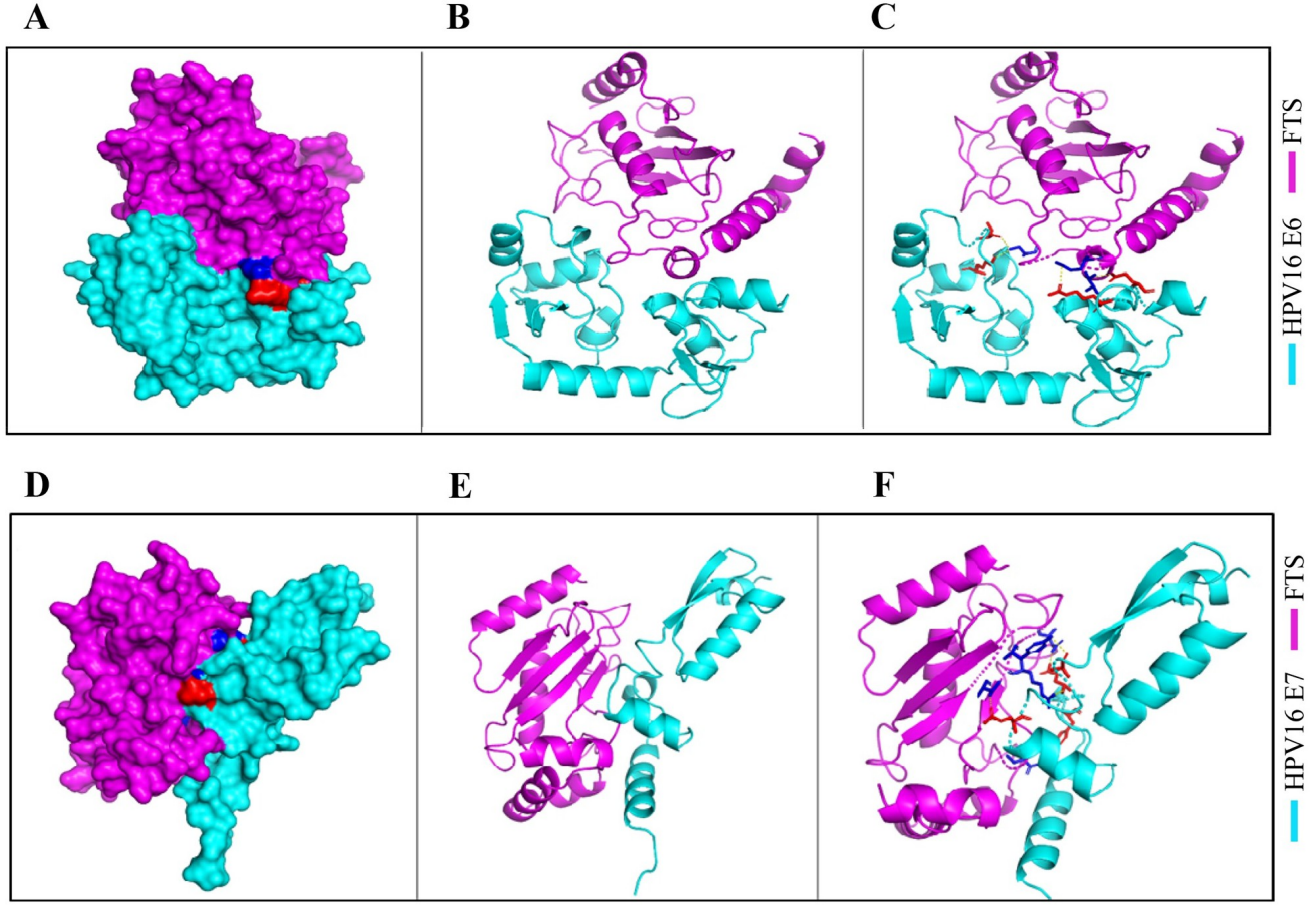
Molecular docking and 3D simulation identify the residues involved in the interaction of FTS with E6/E7. Docking conformation of the HPV16 E6/FTS (A) and E7/FTS (D), interacting residues of the HPV16 E6/FTS (B) and E7/FTS (E) protein-protein complex depicted in sticks representation, three intermolecular H-bonds present in the interface of the complex illustrated by yellow dotted lines, HPV16 E6/FTS (C), and E7/FTS (F). In all the cases, the HPV16 E6/E7 and FTS are represented in cyan and magenta colors, respectively.

We have obtained protein-protein complexes by performing molecular docking studies using the GRAMM-X server. Interacting residues were identified by analyzing the docked complex of the proteins. The Fig 6 represents different types of visualizations of interactions between the proteins using PyMol. The left panel (Fig 6A and 6D) shows the surface representation of the protein-protein docking complex. The right panel (Fig 6C and 6F) shows cartoon representation of docking complex with interacting residues are viewed as sticks representation. The middle panel (Fig 6B and 6E) shows interface residues in cartoon representation.

The docking complex analysis shows unique intermolecular hydrogen bond interactions between proteins, crucial for the binding complex. Interactions between HPV16 E6 and FTS proteins (Fig 6A–6C) are as follows: first H-bond between residues ARG-10 of HPV16 E6 with GLU-203 of FTS with a distance of 3.2 Å. Second H-bond between residues GLN-14 of HPV16 E6 with VAL-200 of FTS with a distance of 3.0 Å. The last two H-bonds are between residues SER-97 and LYS-115 of HPV16 E6 with SER-192 of FTS, with a distance of 3.3 Å and 3.0 Å, respectively. Interactions between HPV16 E7 and FTS proteins (Fig 6D–6F) are as follows: it is found that TYR-25 of HPV16 E7 interacts with ASP-152 of FTS via H-bond with a distance of 2.5 Å. GLN-27 of HPV16 E7 is interacting with ASP-136 and ARG-139 of FTS via H-bond with a distance of 2.8 Å and 3.3 Å. GLN-44 of HPV16 E7 is having H-bond with THR-125 of FTS with a distance of 3.1 Å. And the last H-bond interaction is found between the residues of ALA-50 and TYR-127 with a distance of 3.3 Å.

## Discussion

In the present study, first we observed basal expression of FTS and HPV16 E6 and E7 in the HPV16-positive cervical cancer cell lines, CaSki and SiHa, and demonstrated their co-localization and physical interaction in cervical cancer cell lines and tissues. HPV16 E6 and E7 proteins are oncoplayers [23], critically involved in the onset and progression of cervical cancer. Knockdown of E6 and E7 by RNA interference has been reported to suppress cervical cancer cell growth [24]. The tumor suppressor proteins, p53 and pRb, are the main targets of E6 and E7, respectively, and are often downregulated in cervical cancer [25–27]. In this study, we report that silencing of FTS, or E6 and E7, elevates the levels of p53 and pRb. HPV16 E6 interacts with E6-targeted protein 1, p300/CBP (cAMP response element-binding protein), interferon regulatory factor-3, alteration/deficiency in activation protein 3, G protein-coupled receptor proteolysis site 2, and human minichromosome maintenance-7. All these proteins are involved in DNA replication and transcription regulation. In addition, E6 interacts with E6-associated protein (E6AP), an ubiquitin ligase. In HPV-infected cells, E6AP is hijacked by E6 to target and ubiquitylate cellular proteins for degradation by the UPS. To degrade p53, E6 binds to the LXXLL consensus sequence in the conserved domain of E6AP [25]. In addition, E6 inhibits p53 phosphorylation, thereby preventing p53 binding to the p21 promoter, and thus restraining its cell growth inhibitory functions [28]. Concurrently, E7 binds to pRb via the LXCXE motif and inhibits the interaction of pRb with E2F transcription factors, resulting in the release of transcriptionally active E2F factors. Consequently, the cell is forced to proceed with replication despite an insufficient pool of nucleotides [27]. It has been reported that proliferating cell nuclear antigen (PCNA) enhancement, in addition to p53 and p21 suppression, leads to the activation of E2F1 and relief of inhibition of PCNA, enabling regulation of the cell cycle in breast cancer cells [29]. E7 protein antagonizes DNA sensing and suppresses host immune responses post-infection via the same motif [26]. Additionally, HPV16 E7 interacts with the DREAM (dimerization partner, pRb-like, E2F4, and MuyB) complex, which helps suppress the activity of cell cycle-related genes when not required. Hyper-phosphorylated pRb also plays a tumor-suppressive role by binding Sin1, to inhibit mammalian target of rapamycin Complex 2-mediated activation of Akt [30]. Pal et al. [23] reviewed the roles of HPV E6 and E7 oncoproteins in HPV-linked cervical carcinogenesis and discussed how the two oncoproteins manipulate functions of the tumor suppressors.

Previously, our research group analyzed the expression of FTS in cervical cancer tissues with varied pathologies by immunohistochemistry [11]. FTS immunostaining was not detected in the normal epithelium, while CIN and carcinoma samples exhibited a gradual increase in FTS immunostaining as the pathology advanced. Additionally, the expression of tumor suppressor p21 was reciprocal to the FTS expression levels. The expression levels [31], and positivity rates [32], of E6 and E7 increased progressively with the grade of the CIN lesions, with high concordance with the pathological result [33]. In the present study, we observed that FTS silencing reduced the expression of E6 and E7 oncoproteins; consequently, the expression levels of p53, pRb, and their phosphorylated forms, were elevated in both cervical cancer cell lines (Fig 1B and 1C). Similarly, E6 and E7 silencing downregulated FTS expression (Fig 2A and wB). However, E6 and E7 did not interact with FTS at the transcriptional level (Fig 3, S1 and S2 Figs). Phosphoinositide 3-kinase (PI3K)/Akt is another major cancer survival pathway, and both HPV16 E6 and E7, activate the PI3K/AKT pathway [34, 35]. PI3K regulates Akt, which has a broad range of downstream targets. These Akt downstream targets are regulators of cell proliferation, cell growth, cell mobilization, angiogenesis, and cell survival [34, 35]. This pathway has been associated with higher incidences of cancer onset, progression, metastasis, and drug resistance. Silencing of FTS has been shown to reduce phosphorylation of Akt [12, 13]; in the present study it was also observed that E6 and E7 silencing downregulated FTS and the downstream Akt protein, as well as p-Akt, simultaneously (Fig 2A and 2B). Reduced levels of p-Akt indicate increased susceptibility of cells to apoptotic cell death [36].

To confirm the interaction of FTS with E6 and E7, we performed IF and IP assays. These results confirmed that FTS is functionally (Fig 4), and physically (Fig 5), associated with E6 and E7 in cervical cancer cells and tissues. The molecular docking simulation results (Fig 6) were in complete agreement with the IF, IP, and IHC findings of a physical association between FTS and the HPV16 E6 and E7 proteins, and also successfully identified the residues involved in the interaction of FTS with E6 and E7 (Fig 6C and 6F).

We have previously demonstrated that FTS-silenced HeLa cells lost clonogenic potential, and thus their survival rates were significantly lower than those of control HeLa cells [11]. In this study, we also found that FTS-silenced cells had higher expression levels of tumor suppressor proteins, and lower expression levels of cell survival markers (Fig 2). This signifies a role of FTS in HPV-mediated cervical carcinogenesis. The molecular mechanism of E6 and E7 regulation by FTS, or vice versa, is intricate, and remains unelucidated. Our findings suggest that there may be crosstalk between FTS and the E6 and E7 proteins through collaborative regulation in HPV16-mediated cervical cancer. Given the current findings that FTS is linked to the regulation of both HPV16 E6 and E7, we believe that FTS may be involved in molecular pathways common to E6 and E7.

In summary, our data demonstrated an association between FTS and HPV16 E6 and E7 expression. Interestingly, silencing of FTS led to downregulation of E6 and E7, and increased levels of tumor suppressor proteins, as well as their phosphorylated forms. The present study reports a regulatory association of FTS with E6 and E7. This study further warrants to investigate phenotypes of E6 and E7 in FTS overexpressing cells and examine *in vivo* tumor formation capabilities of FTS silenced cervical cancer cells. Although our findings cannot fully explain the mechanism of interaction between FTS and HPV16 E6 and E7, it presents a necessary prelude and clearly show that FTS may play an important role in the initiation and progression of uterine cervical cancer. We conclude, FTS is a potential target for the prevention of cervical cancer development and progression.

## Supporting information

S1 File (PPTX)

S1 Fig CaSki and SiHa cells grown in 60 mm culture plate were treated with 0.5 μM MG262 (#I-120, Boston Biochem, USA) After 24 h of MG262 treatment, the cells were lysed and western blotting for E6, E7 and FTS was performed as described in material and methods. It can be seen that MG262 abolishes FTS silencing effects and rescues E6/E7 proteins from degradation by at least 50% in both the cell lines.
(TIF)

S2 Fig Cells were treated with 100 μM cycloheximide (CHX; #239763, Millipore, USA), in a time dependent manner. After indicated time period, the cells were lysed and western blotting was performed for E6, E7 and FTS, as described in materials and methods. We can see that E6 and E7 proteins are reduced in response to CHX treatment in a time dependent manner. In FTS silenced cells, the depletion of these proteins is faster. It’s remarkable to note that in FTS intact cells, E6 protein takes 6 h in CaSki and 1 h in SiHa cells to completely diminish, however in FTS silenced cells it takes only 1 h. Even though FTS intact and FTS silenced SiHa cells do not show traces of E6 protein after 1 h, we can see that the protein levels are quite low (~50%) in FTS silenced cells as compared to FTS intact cells. E7 protein doesn’t show any drastic changes in expression levels but the time dependent effect is evident, however 24 h was not enough for its complete degradation. This probably means that E6 has higher protection than E7 from proteasomal degradation due to FTS interaction with these proteins. Considering that there was no change in these targets at the transcription level we can say that FTS interacts with these targets post-translationally and prevents their degradation by proteasomes.
(TIF)

S1 Table Pearson’s coefficient for co-localization of FTS with E6 or E7 in HPV16 positive CaSki and SiHa cell lines. A value of 1.0 means 100% co-localization.
(DOCX)
