# Supplementary material for: Fused toes homolog, a potential molecular regulator of human papillomavirus type 16 E6 and E7 oncoproteins in cervical cancer
Source: PLoS One. 2022 Apr 14;17(4):e0266532. doi: 10.1371/journal.pone.0266532 (PMC9009596; doi:10.1371/journal.pone.0266532)
Supplement: S1 File — (PPTX) [file pone.0266532.s001.pptx]

## Slide 1
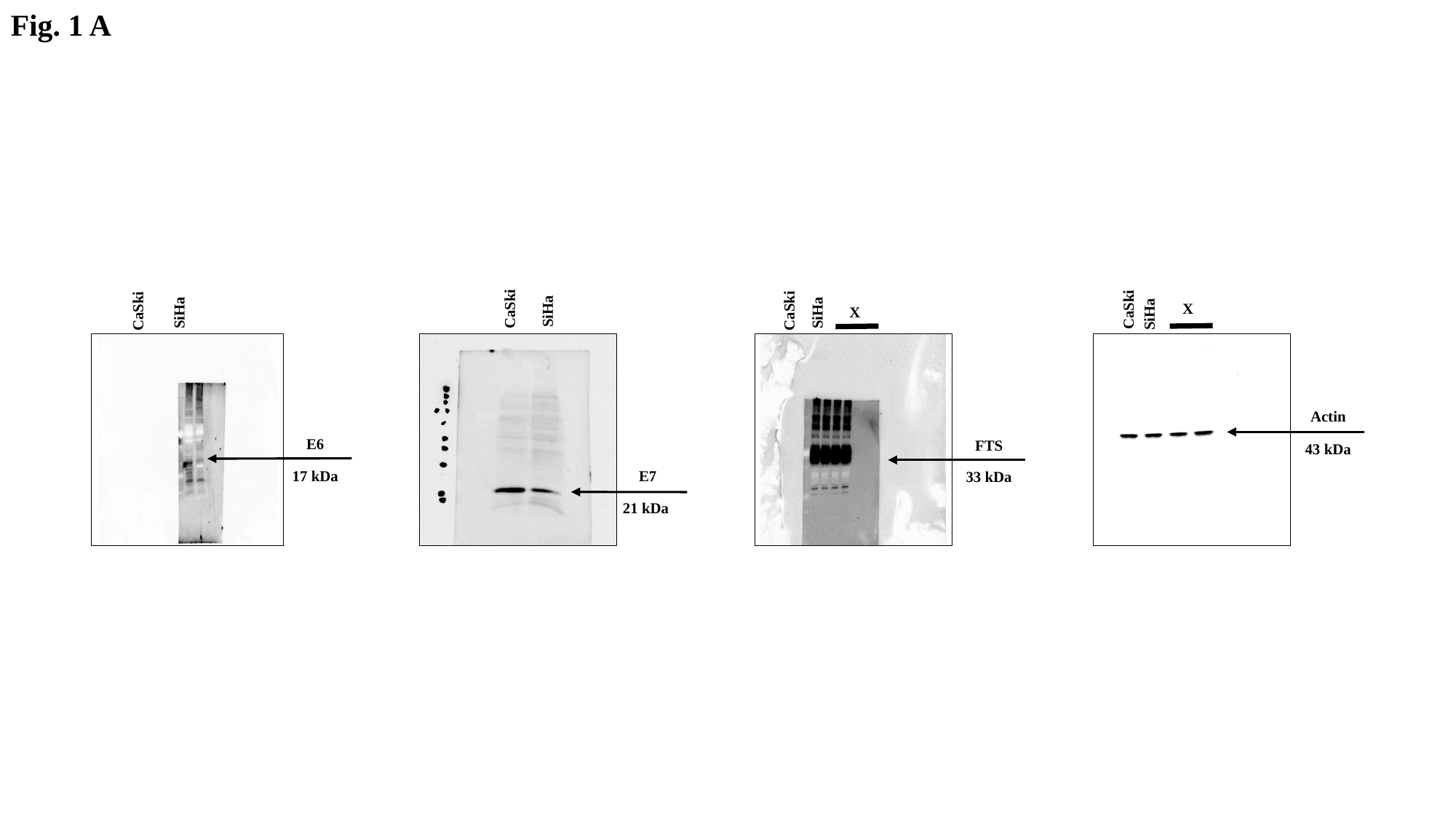

Fig. 1 A
SiHa
CaSki
E7
21 kDa
SiHa
CaSki
E6
17 kDa
SiHa
CaSki
X
FTS
33 kDa
CaSki
X
SiHa
Actin
43 kDa

## Slide 2
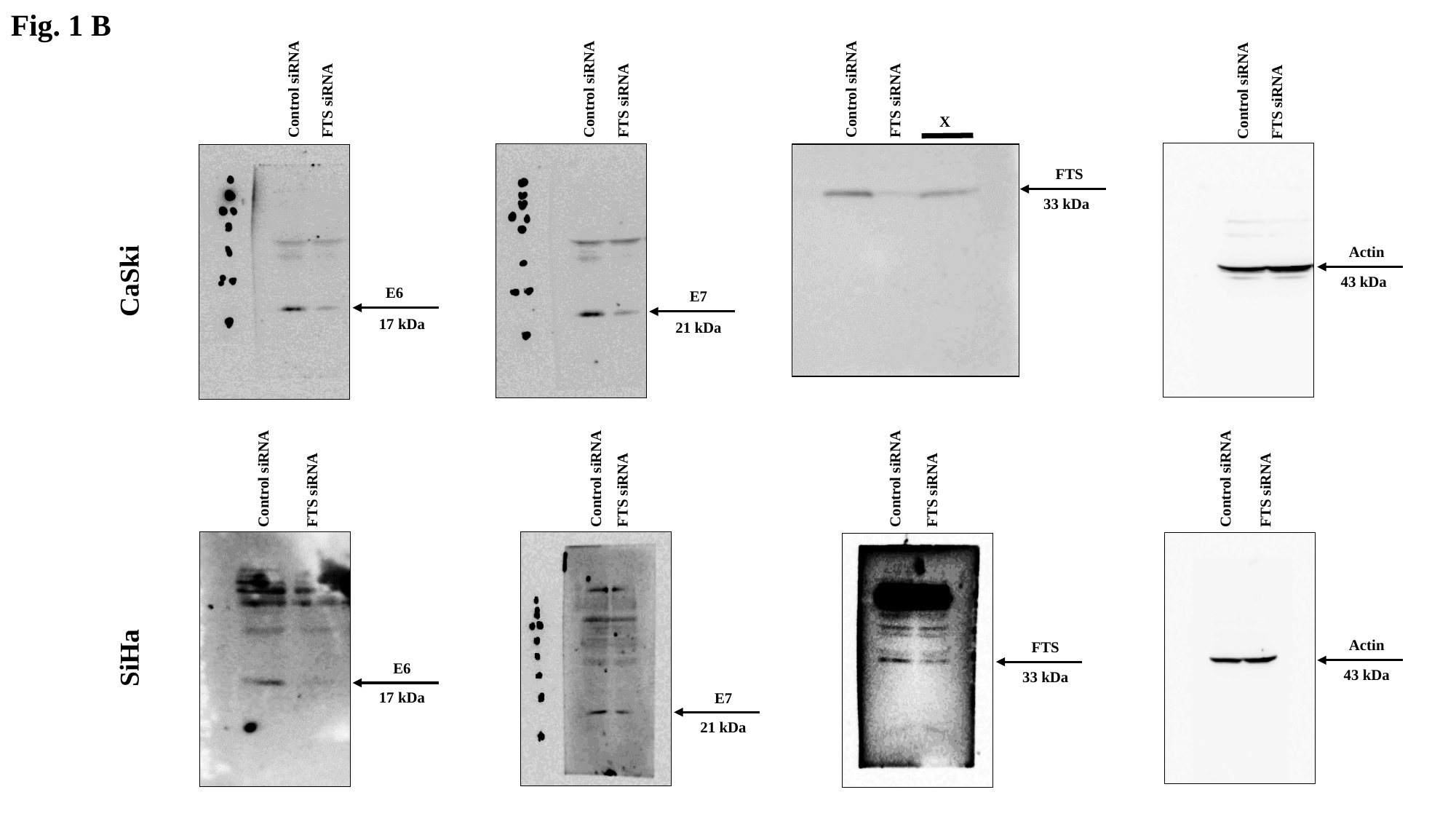

Fig. 1 B
Control siRNA
FTS siRNA
X
FTS
33 kDa
Control siRNA
FTS siRNA
E6
17 kDa
Control siRNA
FTS siRNA
E7
21 kDa
Control siRNA
FTS siRNA
Actin
43 kDa
CaSki
Control siRNA
FTS siRNA
E6
17 kDa
Control siRNA
FTS siRNA
E7
21 kDa
Control siRNA
FTS siRNA
FTS
33 kDa
Control siRNA
FTS siRNA
Actin
43 kDa
SiHa

## Slide 3
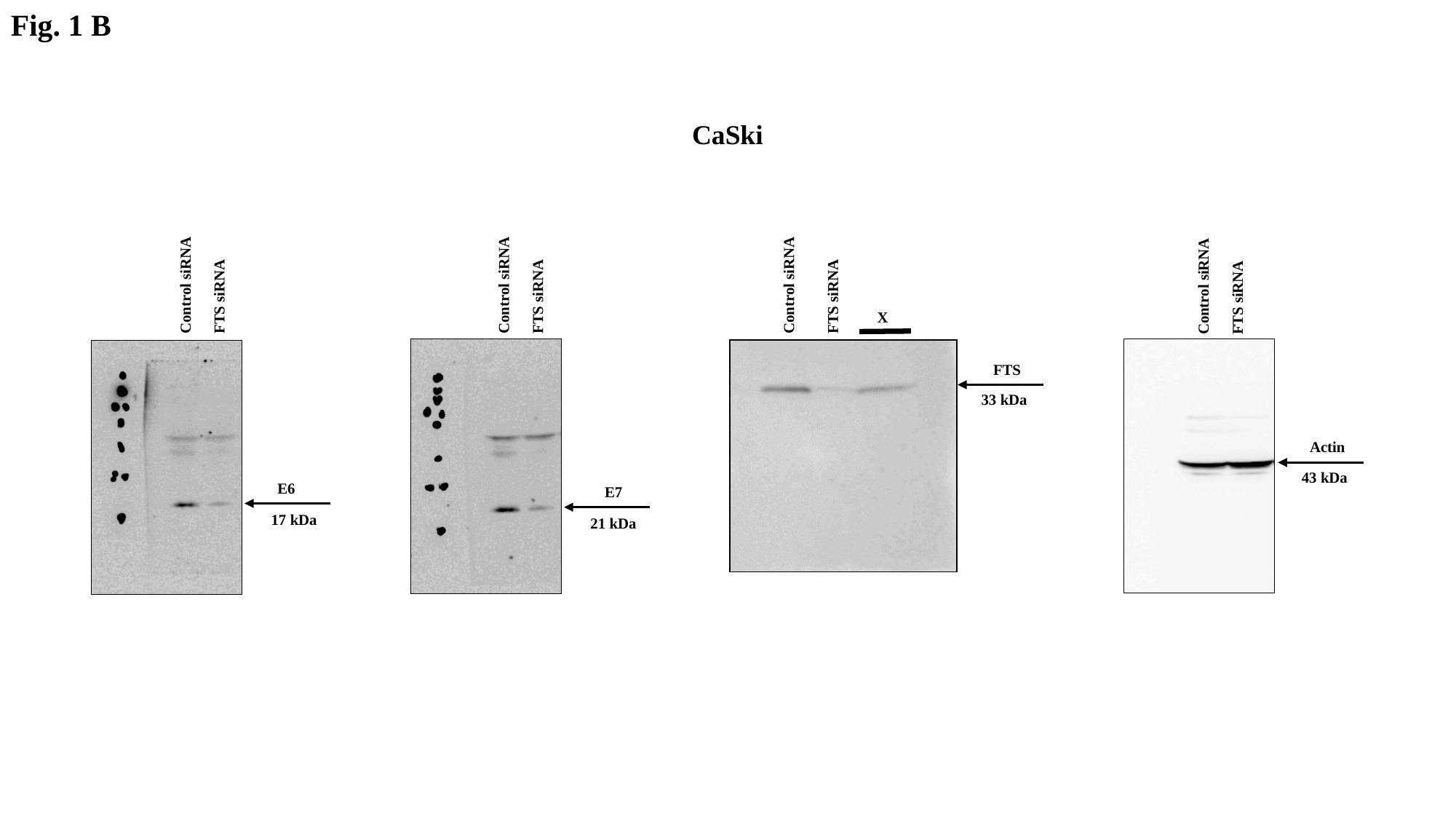

Fig. 1 B
CaSki
Control siRNA
FTS siRNA
E6
17 kDa
Control siRNA
FTS siRNA
E7
21 kDa
Control siRNA
FTS siRNA
X
FTS
33 kDa
Control siRNA
FTS siRNA
Actin
43 kDa

## Slide 4
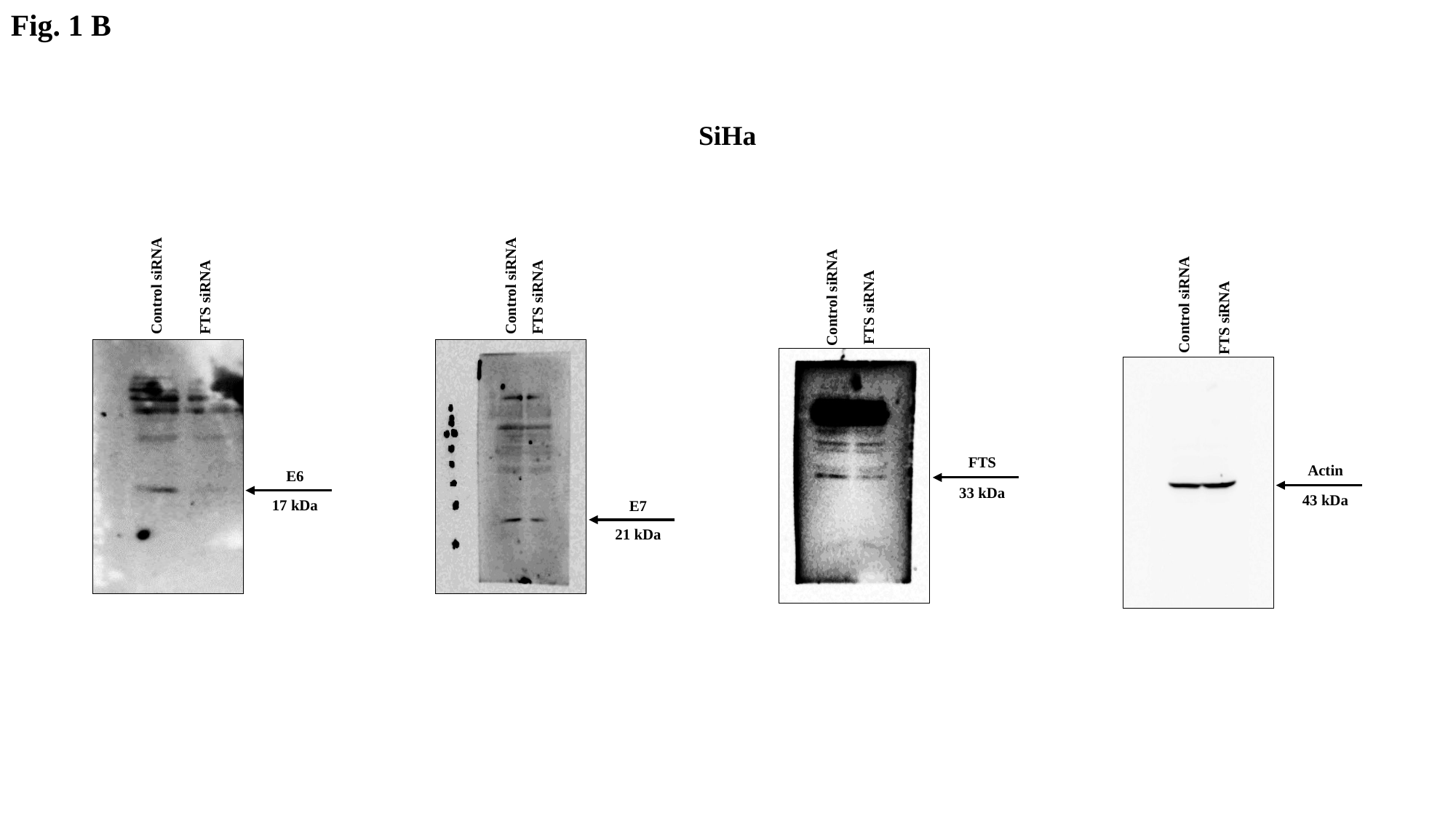

Fig. 1 B
SiHa
Control siRNA
FTS siRNA
E6
17 kDa
Control siRNA
FTS siRNA
E7
21 kDa
Control siRNA
FTS siRNA
FTS
33 kDa
Control siRNA
FTS siRNA
Actin
43 kDa

## Slide 5
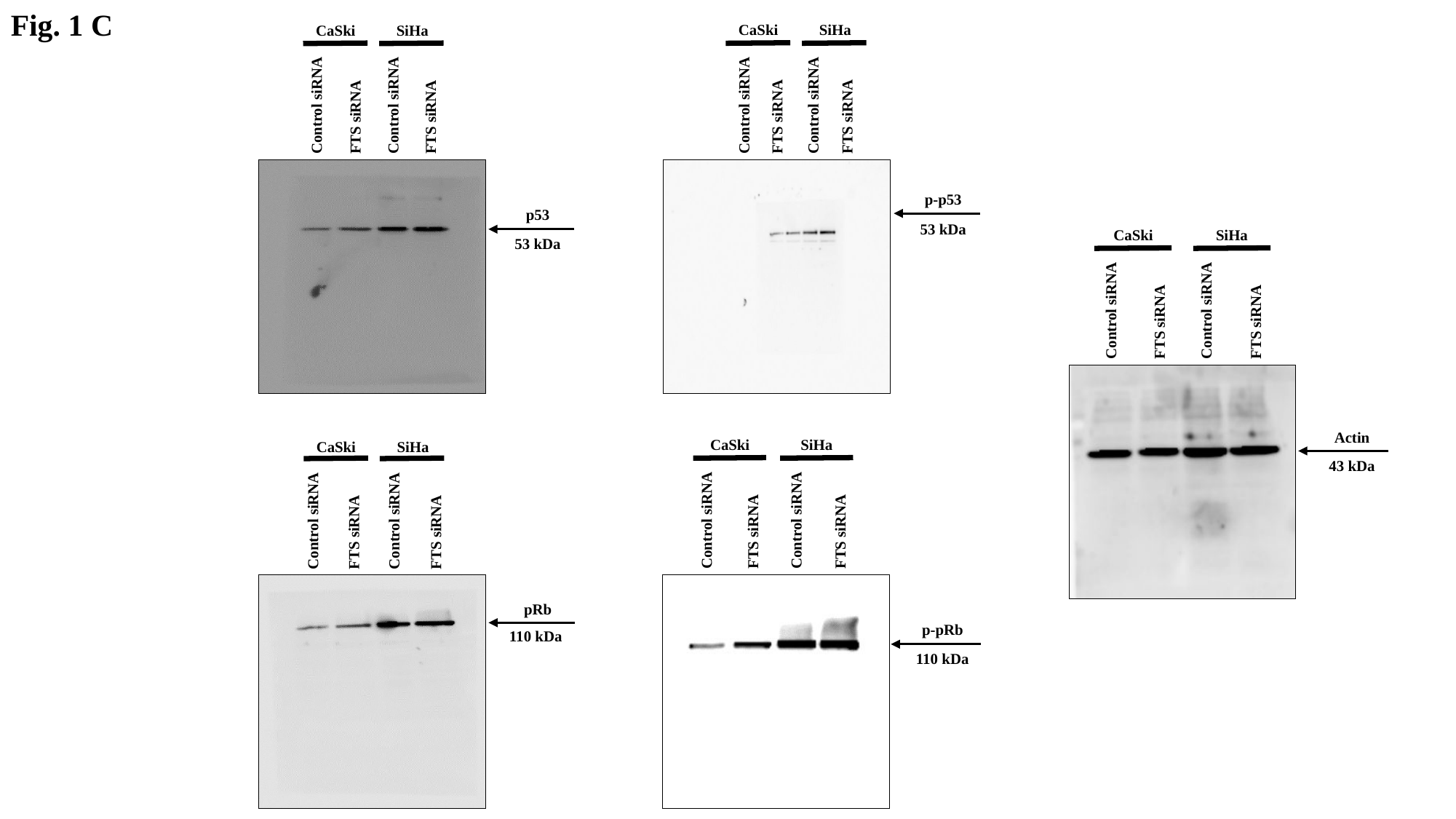

Fig. 1 C
CaSki
SiHa
Control siRNA
FTS siRNA
Control siRNA
FTS siRNA
p-p53
53 kDa
CaSki
SiHa
Control siRNA
FTS siRNA
Control siRNA
FTS siRNA
p53
53 kDa
CaSki
SiHa
Control siRNA
FTS siRNA
Control siRNA
FTS siRNA
Actin
43 kDa
CaSki
SiHa
Control siRNA
FTS siRNA
Control siRNA
FTS siRNA
p-pRb
110 kDa
CaSki
SiHa
Control siRNA
FTS siRNA
Control siRNA
FTS siRNA
pRb
110 kDa

## Slide 6
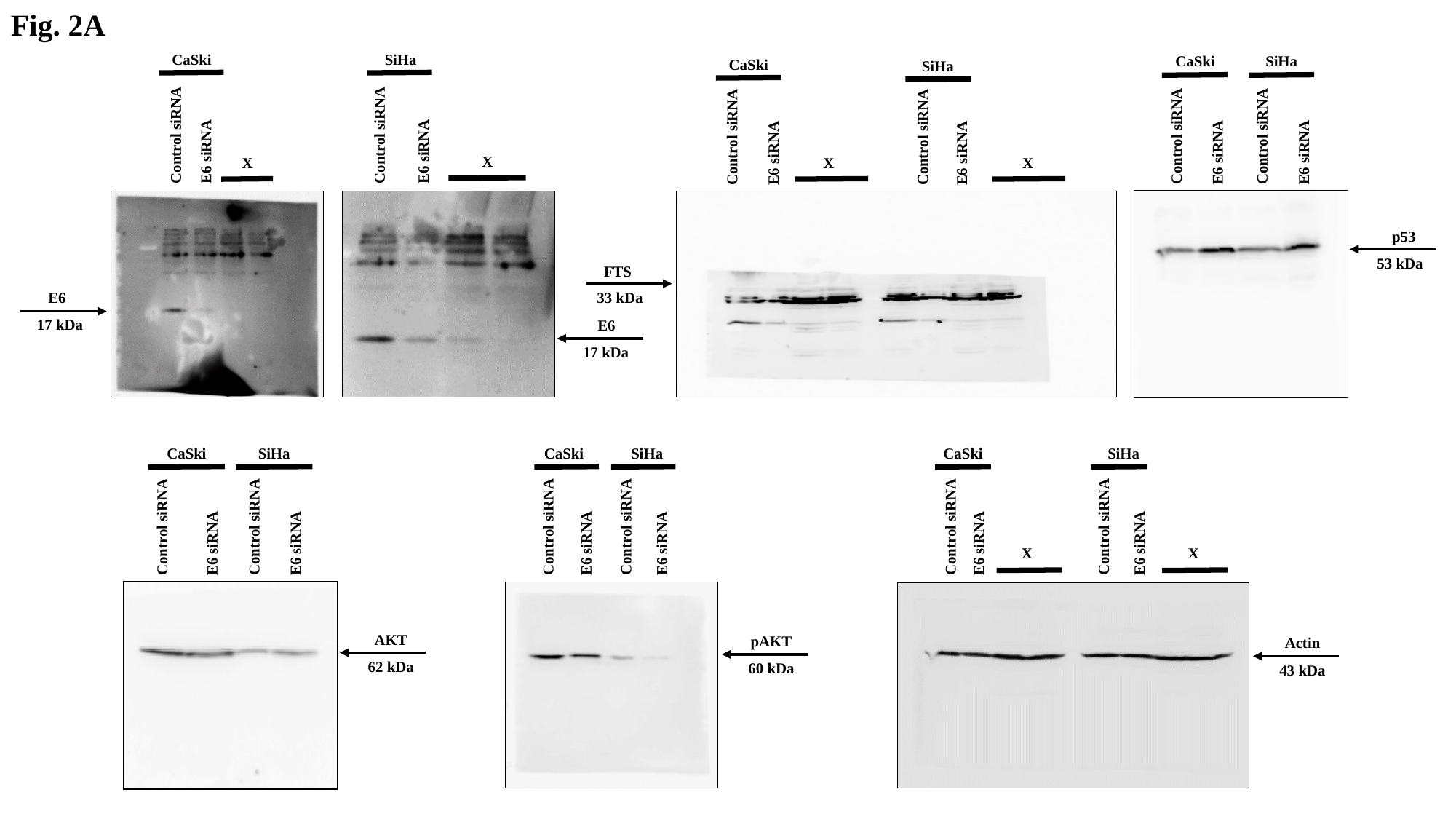

Fig. 2A
CaSki
SiHa
Control siRNA
Control siRNA
E6 siRNA
E6 siRNA
X
X
FTS
33 kDa
CaSki
Control siRNA
E6 siRNA
X
E6
17 kDa
SiHa
Control siRNA
E6 siRNA
X
E6
17 kDa
CaSki
SiHa
Control siRNA
E6 siRNA
Control siRNA
E6 siRNA
p53
53 kDa
CaSki
SiHa
Control siRNA
E6 siRNA
Control siRNA
E6 siRNA
AKT
62 kDa
CaSki
SiHa
Control siRNA
E6 siRNA
Control siRNA
E6 siRNA
pAKT
60 kDa
CaSki
SiHa
Control siRNA
E6 siRNA
Control siRNA
E6 siRNA
X
X
Actin
43 kDa

## Slide 7
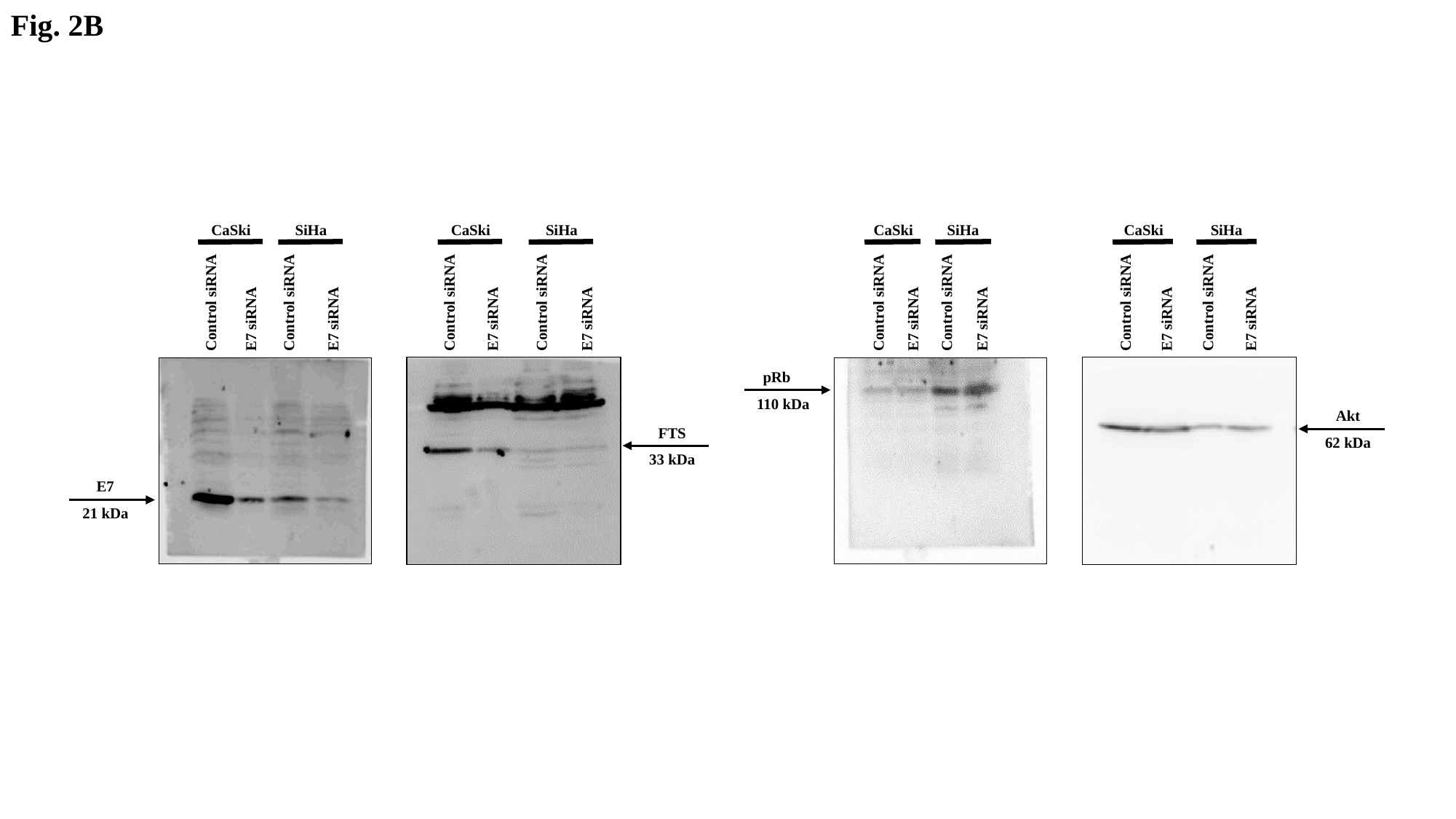

Fig. 2B
CaSki
SiHa
Control siRNA
E7 siRNA
Control siRNA
E7 siRNA
E7
21 kDa
CaSki
SiHa
Control siRNA
E7 siRNA
Control siRNA
E7 siRNA
FTS
33 kDa
CaSki
SiHa
Control siRNA
E7 siRNA
Control siRNA
E7 siRNA
pRb
110 kDa
CaSki
SiHa
Control siRNA
E7 siRNA
Control siRNA
E7 siRNA
Akt
62 kDa

## Slide 8
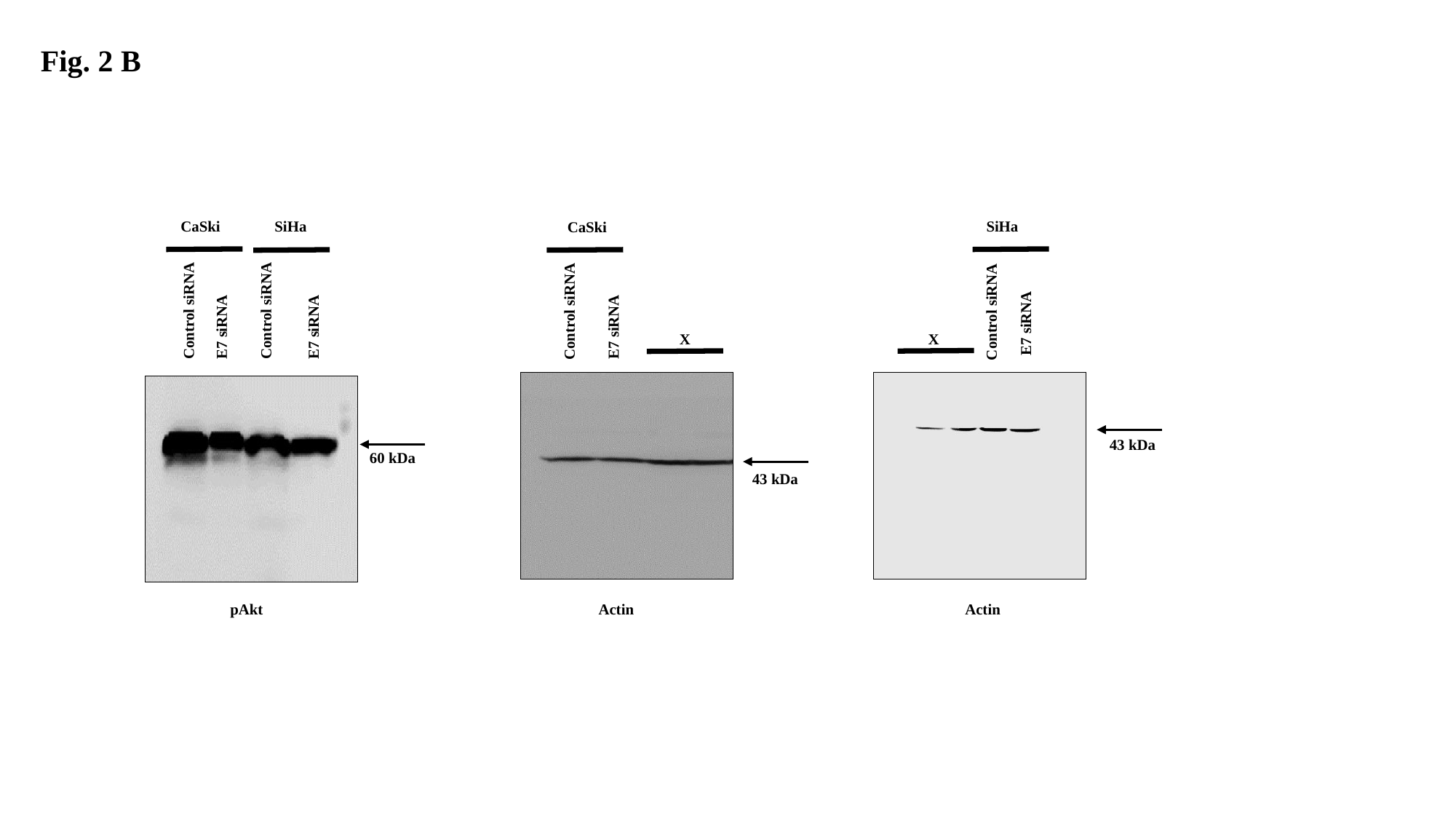

Fig. 2 B
SiHa
SiHa
CaSki
CaSki
Control siRNA
Control siRNA
Control siRNA
Control siRNA
E7 siRNA
E7 siRNA
E7 siRNA
E7 siRNA
X
X
43 kDa
60 kDa
43 kDa
pAkt
Actin
Actin

## Slide 9
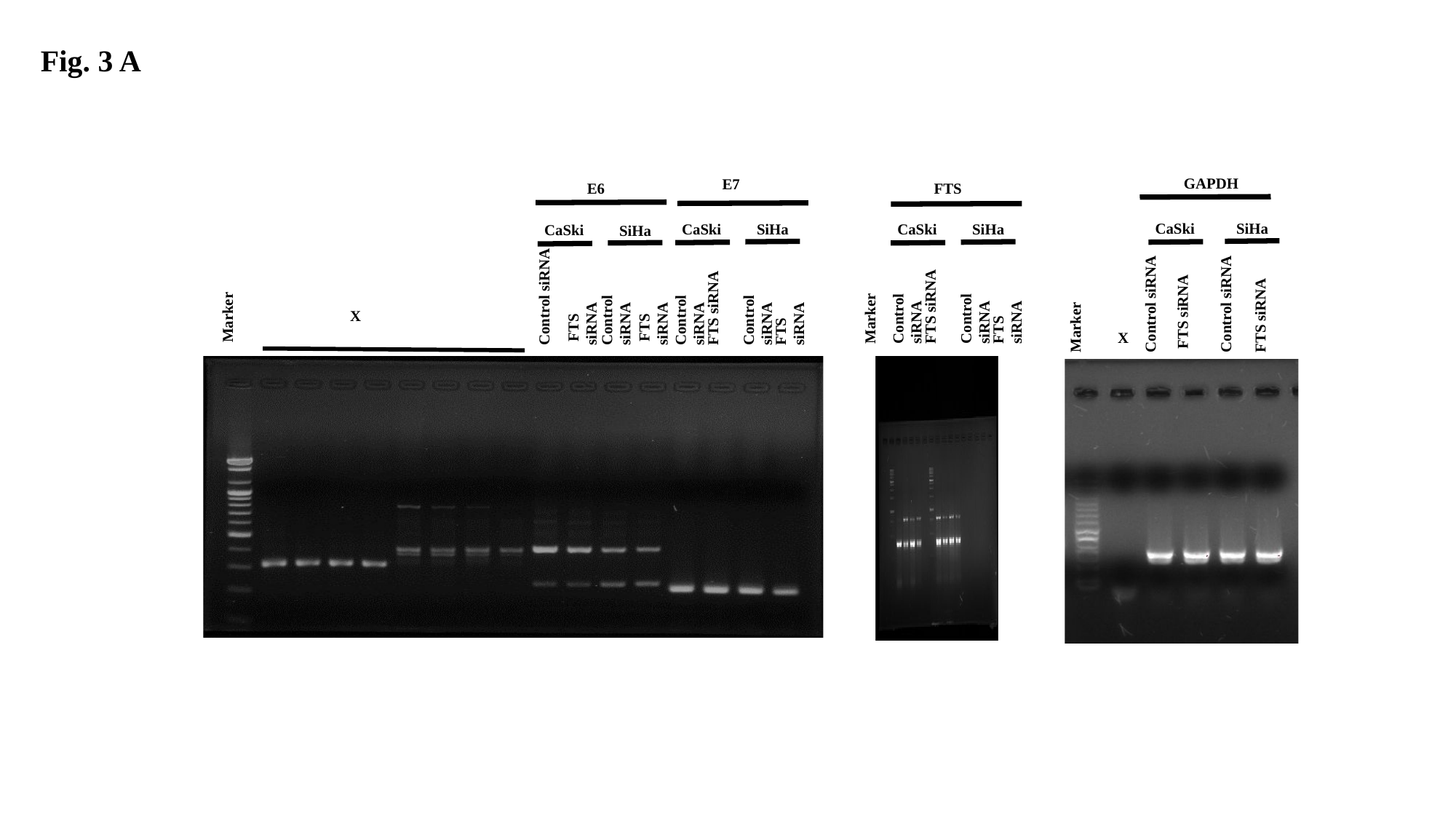

Fig. 3 A
GAPDH
E7
E6
FTS
Marker
Marker
Marker
CaSki
SiHa
CaSki
SiHa
CaSki
SiHa
CaSki
SiHa
Control siRNA
Control siRNA
Control siRNA
Control siRNA
Control siRNA
Control siRNA
Control siRNA
FTS siRNA
FTS siRNA
Control siRNA
FTS siRNA
FTS siRNA
FTS siRNA
 FTS siRNA
 FTS siRNA
 FTS siRNA
X
X

## Slide 10
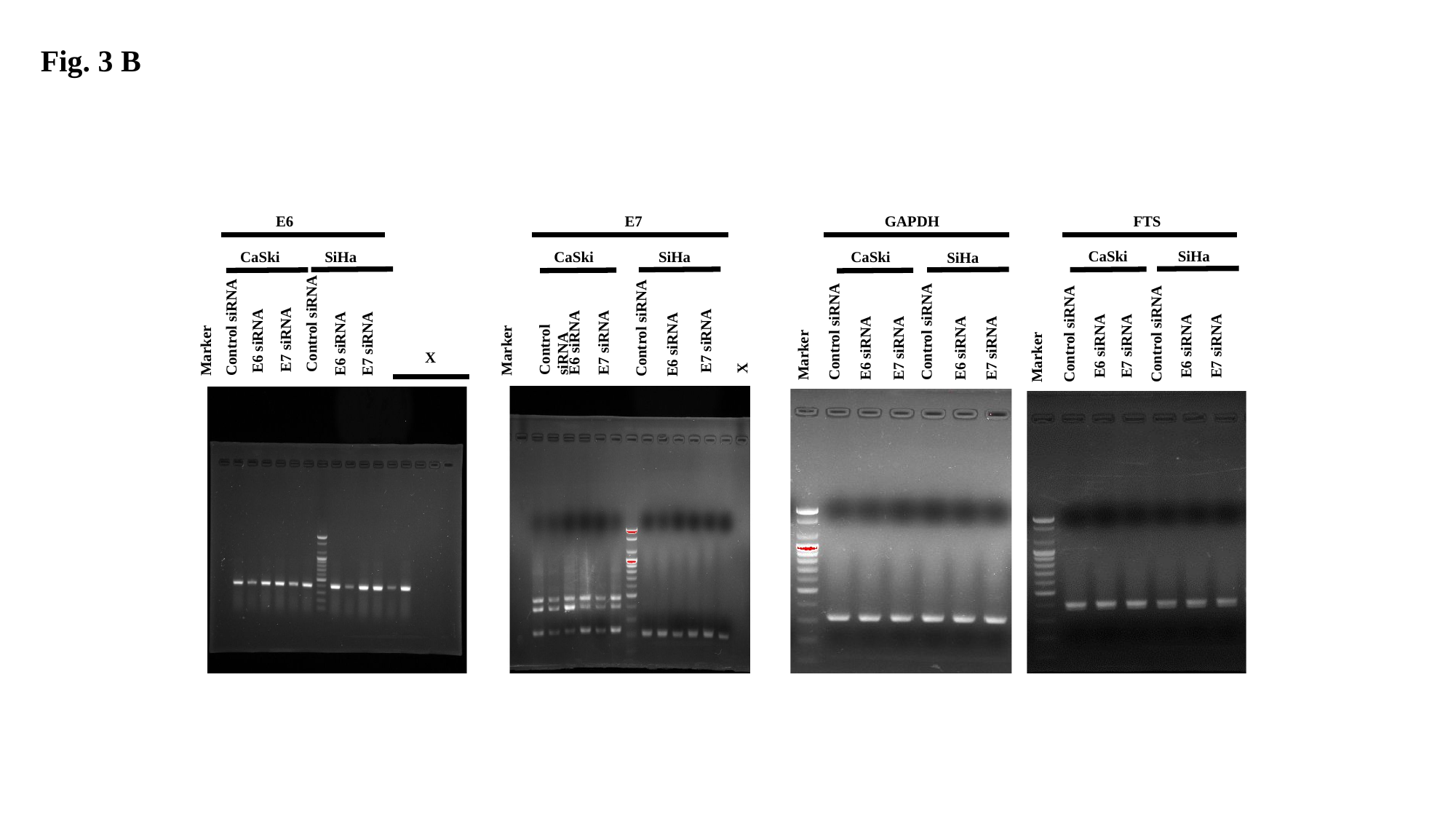

Fig. 3 B
Control siRNA
Control siRNA
Control siRNA
E6
E7
GAPDH
FTS
Control siRNA
 Control siRNA
 E6 siRNA
Control siRNA
E6 siRNA
 E7 siRNA
E7 siRNA
E7 siRNA
Marker
 E7 siRNA
CaSki
SiHa
CaSki
SiHa
CaSki
E7 siRNA
CaSki
SiHa
SiHa
 E6 siRNA
E6 siRNA
E6 siRNA
 E7 siRNA
Control siRNA
 E6 siRNA
Control siRNA
E7 siRNA
E6 siRNA
 E7 siRNA
E6 siRNA
X
Marker
Marker
Marker
X

## Slide 11
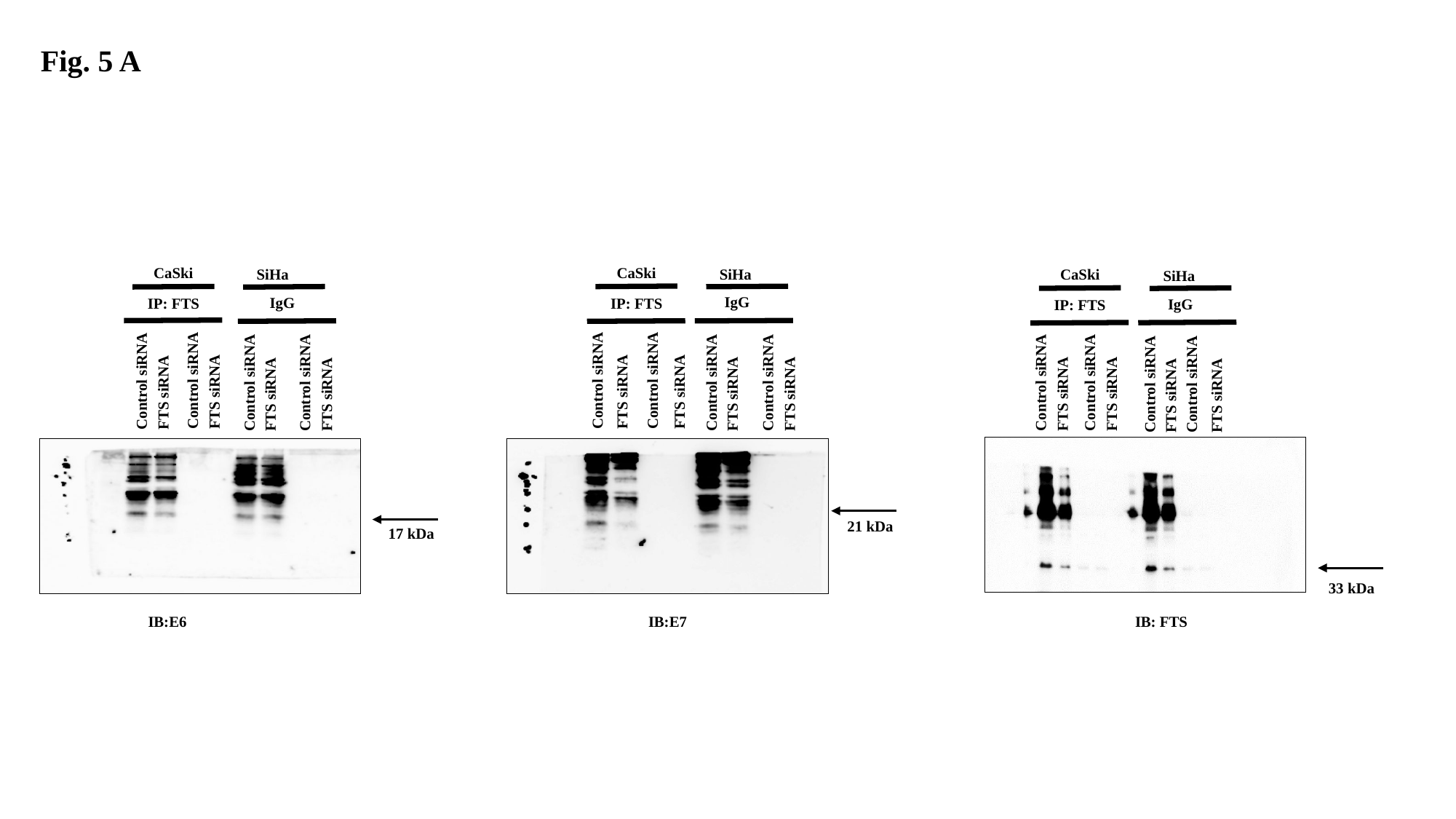

Fig. 5 A
CaSki
CaSki
SiHa
SiHa
CaSki
SiHa
IgG
IgG
IP: FTS
IP: FTS
IgG
IP: FTS
Control siRNA
Control siRNA
Control siRNA
Control siRNA
Control siRNA
Control siRNA
Control siRNA
Control siRNA
Control siRNA
Control siRNA
Control siRNA
Control siRNA
FTS siRNA
FTS siRNA
FTS siRNA
FTS siRNA
FTS siRNA
FTS siRNA
FTS siRNA
FTS siRNA
FTS siRNA
FTS siRNA
FTS siRNA
FTS siRNA
21 kDa
17 kDa
33 kDa
IB:E6
IB:E7
IB: FTS

## Slide 12
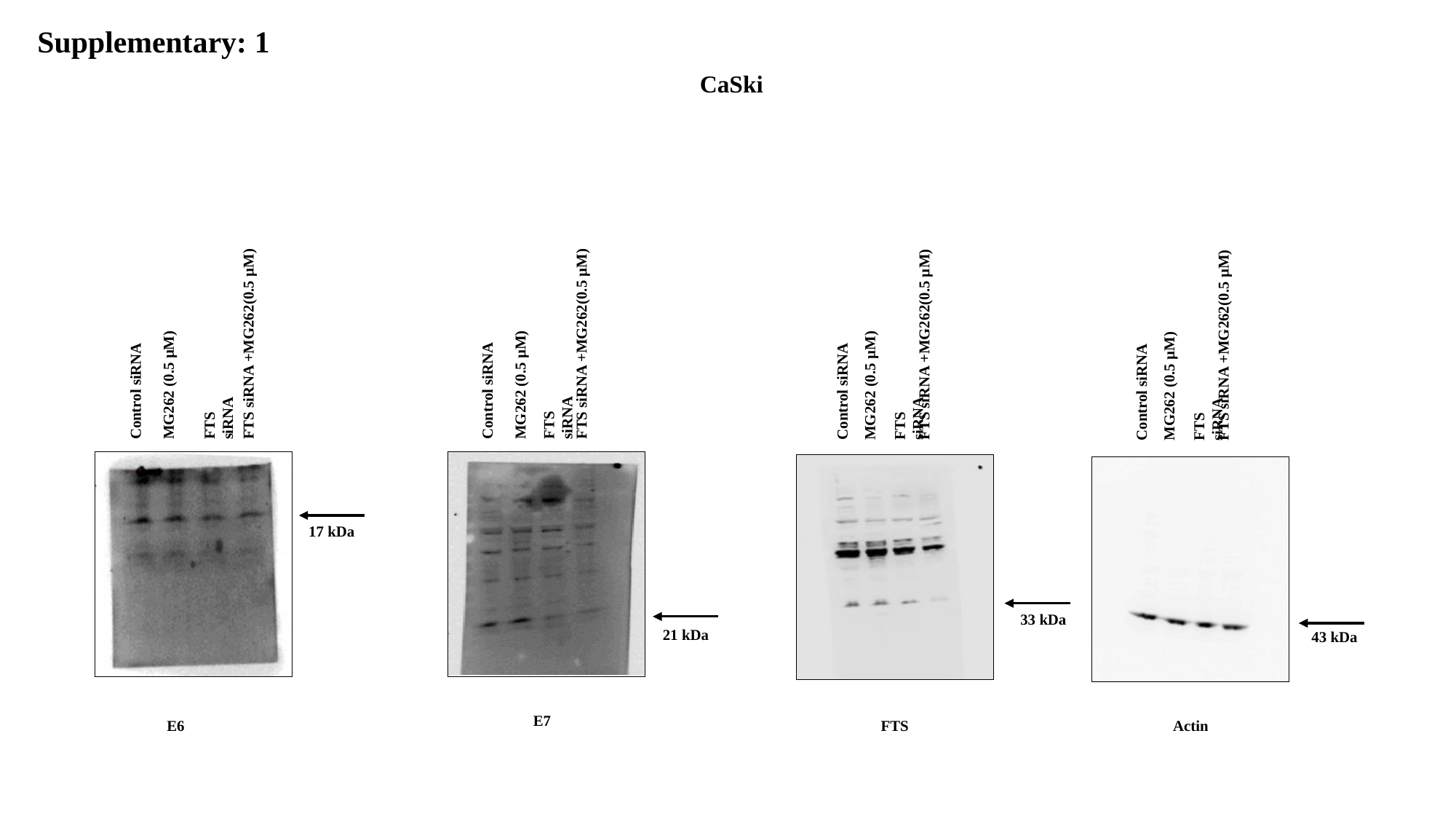

Supplementary: 1
CaSki
FTS siRNA +MG262(0.5 µM)
FTS siRNA +MG262(0.5 µM)
FTS siRNA +MG262(0.5 µM)
FTS siRNA +MG262(0.5 µM)
MG262 (0.5 µM)
MG262 (0.5 µM)
MG262 (0.5 µM)
MG262 (0.5 µM)
Control siRNA
Control siRNA
Control siRNA
Control siRNA
FTS siRNA
FTS siRNA
FTS siRNA
FTS siRNA
17 kDa
33 kDa
21 kDa
43 kDa
E7
E6
FTS
Actin

## Slide 13
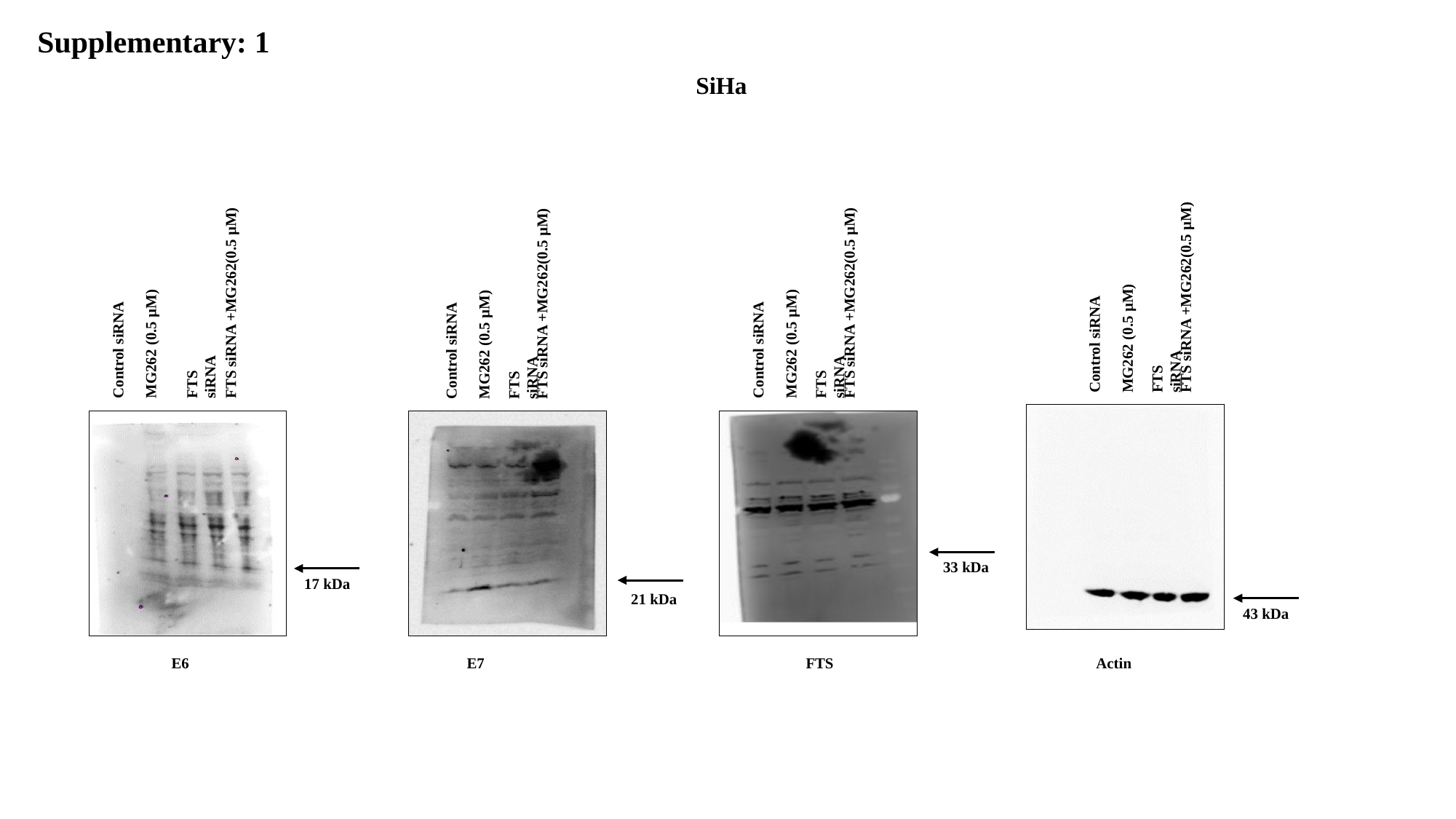

Supplementary: 1
SiHa
FTS siRNA +MG262(0.5 µM)
FTS siRNA +MG262(0.5 µM)
FTS siRNA +MG262(0.5 µM)
FTS siRNA +MG262(0.5 µM)
MG262 (0.5 µM)
MG262 (0.5 µM)
MG262 (0.5 µM)
MG262 (0.5 µM)
Control siRNA
Control siRNA
Control siRNA
Control siRNA
FTS siRNA
FTS siRNA
FTS siRNA
FTS siRNA
33 kDa
17 kDa
21 kDa
43 kDa
E6
E7
FTS
Actin

## Slide 14
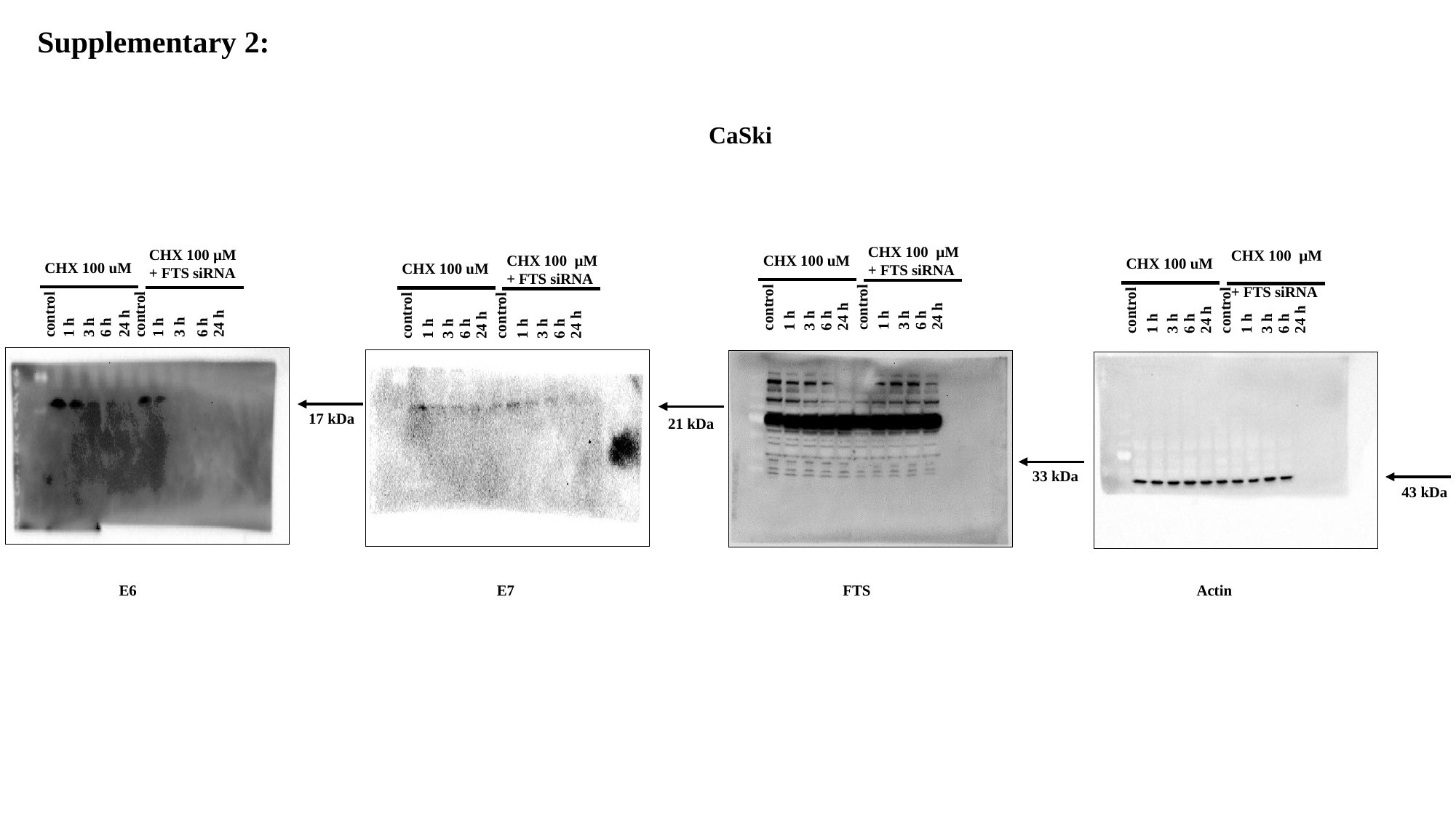

Supplementary 2:
CaSki
1 h
3 h
24 h
control
6 h
1 h
3 h
24 h
control
6 h
1 h
3 h
24 h
control
6 h
1 h
3 h
24 h
control
6 h
CHX 100 µM
+ FTS siRNA
1 h
3 h
24 h
control
6 h
1 h
3 h
24 h
control
6 h
CHX 100 µM
+ FTS siRNA
1 h
3 h
24 h
control
6 h
1 h
3 h
24 h
control
6 h
CHX 100 µM
+ FTS siRNA
CHX 100 µM + FTS siRNA
CHX 100 uM
CHX 100 uM
CHX 100 uM
CHX 100 uM
17 kDa
21 kDa
33 kDa
43 kDa
E6
E7
FTS
Actin

## Slide 15
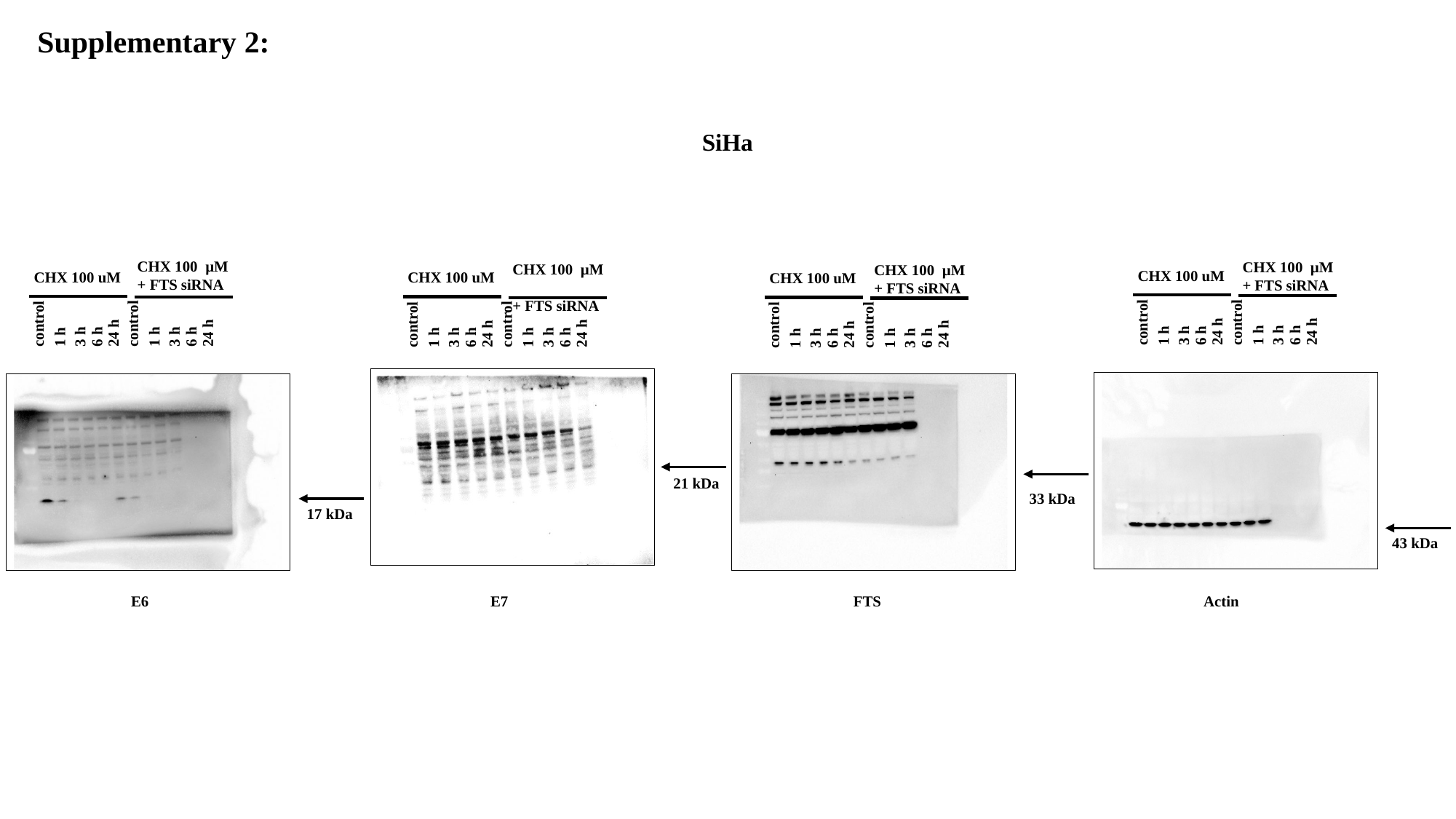

Supplementary 2:
SiHa
1 h
3 h
24 h
control
6 h
1 h
3 h
24 h
control
6 h
1 h
3 h
24 h
control
6 h
1 h
3 h
24 h
control
6 h
1 h
3 h
24 h
control
6 h
1 h
3 h
24 h
control
6 h
1 h
3 h
24 h
control
6 h
1 h
3 h
24 h
control
6 h
CHX 100 µM
+ FTS siRNA
CHX 100 µM + FTS siRNA
CHX 100 µM
+ FTS siRNA
CHX 100 µM + FTS siRNA
CHX 100 uM
CHX 100 uM
CHX 100 uM
CHX 100 uM
21 kDa
33 kDa
17 kDa
43 kDa
E6
E7
FTS
Actin
