# Supplementary figures and images for: Fused toes homolog, a potential molecular regulator of human papillomavirus type 16 E6 and E7 oncoproteins in cervical cancer
Source: PLoS One. 2022 Apr 14;17(4):e0266532. doi: 10.1371/journal.pone.0266532 (PMC9009596; doi:10.1371/journal.pone.0266532)

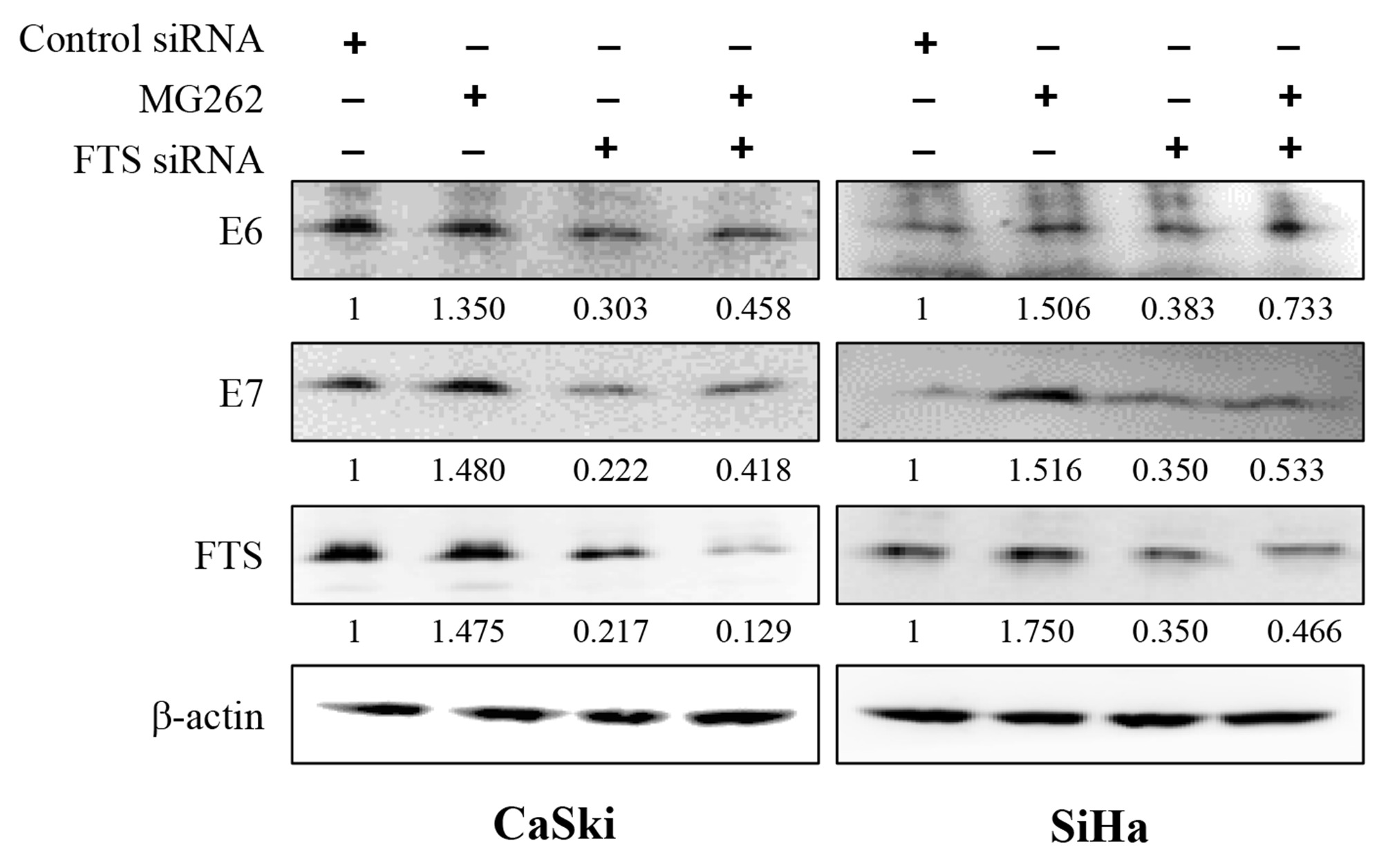

Supplement: S1 Fig — It can be seen that MG262 abolishes FTS silencing effects and rescues E6/E7 proteins from degradation by at least 50% in both the cell lines. (TIF) [file pone.0266532.s002.tif]

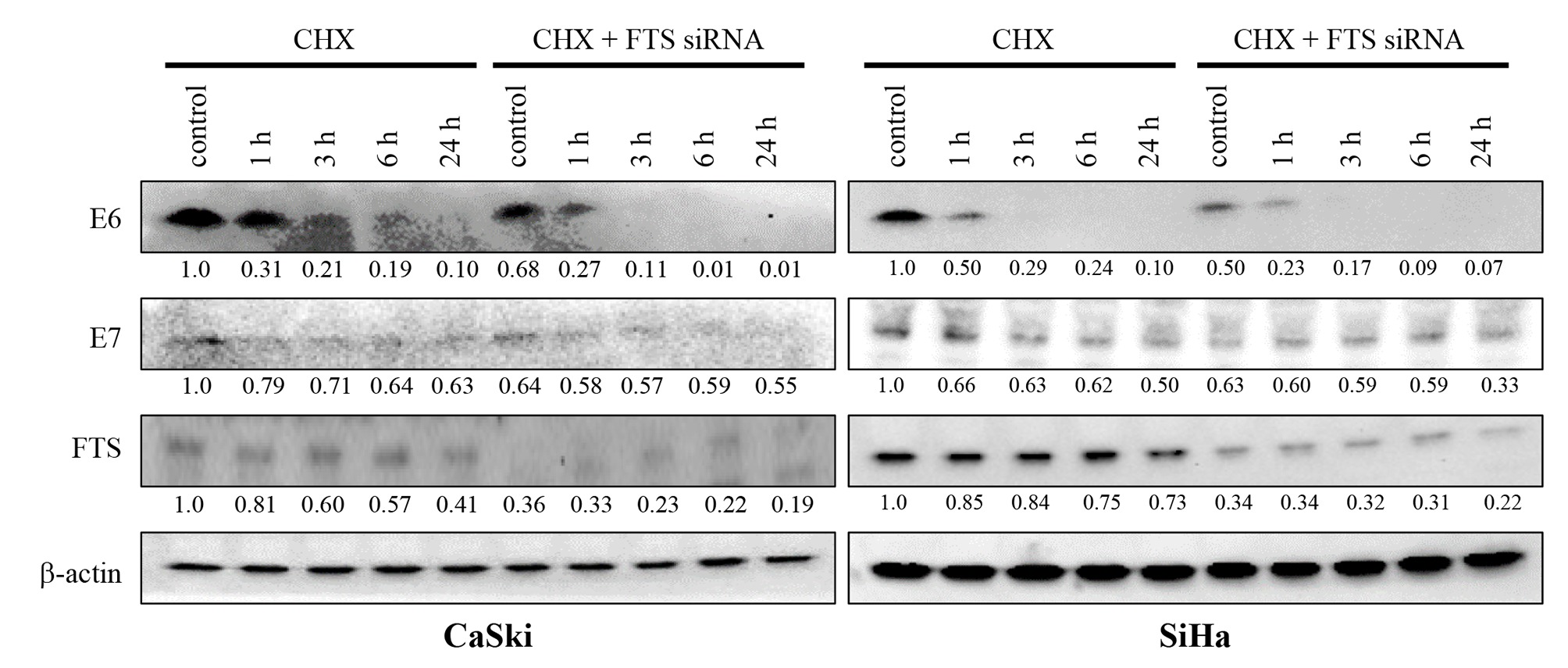

Supplement: S2 Fig — After indicated time period, the cells were lysed and western blotting was performed for E6, E7 and FTS, as described in materials and methods. We can see that E6 and E7 proteins are reduced in response to CHX treatment in a time dependent manner. In FTS silenced cells, the depletion of these proteins is faster. It’s remarkable to note that in FTS intact cells, E6 protein takes 6 h in CaSki and 1 h in SiHa cells to completely diminish, however in FTS silenced cells it takes only 1 h. Even though FTS intact and FTS silenced SiHa cells do not show traces of E6 protein after 1 h, we can see that the protein levels are quite low (~50%) in FTS silenced cells as compared to FTS intact cells. E7 protein doesn’t show any drastic changes in expression levels but the time dependent effect is evident, however 24 h was not enough for its complete degradation. This probably means that E6 has higher protection than E7 from proteasomal degradation due to FTS interaction with these proteins. Considering that there was no change in these targets at the transcription level we can say that FTS interacts with these targets post-translationally and prevents their degradation by proteasomes. (TIF) [file pone.0266532.s003.tif]
