# Supplementary material for: Fused toes homolog, a potential molecular regulator of human papillomavirus type 16 E6 and E7 oncoproteins in cervical cancer
Source: PLoS One. 2022 Apr 14;17(4):e0266532. doi: 10.1371/journal.pone.0266532 (PMC9009596; doi:10.1371/journal.pone.0266532)
Supplement: S1 Table — A value of 1.0 means 100% co-localization. (DOCX) [file pone.0266532.s004.docx]

**S1 Table: Pearson’s coefficient for colocalization of FTS with E6 or E7**

|  |  |  |  | Pearson’s coefficient | FTS over E6/E7 (M1) | E6/E7 over FTS (M2) |
| --- | --- | --- | --- | --- | --- | --- |
| 1 | CaSki | E6 | control | 0.893 | 0.795 | 0.971 |
| 2 |  |  | FTS siRNA | 0.889 | 0.833 | 0.952 |
| 3 |  | E7 | control | 0.928 | 0.883 | 0.974 |
| 4 |  |  | FTS siRNA | 0.845 | 0.762 | 0.873 |
| 5 | SiHa | E6 | control | 0.867 | 0.832 | 0.948 |
| 6 |  |  | FTS siRNA | 0.890 | 0.860 | 0.963 |
| 7 |  | E7 | control | 0.938 | 0.907 | 0.975 |
| 8 |  |  | FTS siRNA | 0.933 | 0.856 | 0.963 |

Pearson’s coefficient for co-localization of FTS with E6 or E7 in HPV16 positive CaSki and SiHa cell lines. A value of 1.0 means 100% colocalization.
